# Targeting KDM3B Elicits Anti‐tumor Immunity by Alleviating SHP1–mediated STING Suppression in Triple–Negative Breast Cancer

**DOI:** 10.1002/advs.75846

**Published:** 2026-05-27

**Authors:** Xiaolong Wang, Wenhao Li, Yifei Wang, Ning Zhang, Bing Chen, Wenjing Zhao, Lijuan Wang, Dan Luo, Qifeng Yang

**Affiliations:** ^1^ Department of Breast Surgery General Surgery Qilu Hospital of Shandong University Jinan Shandong China; ^2^ Biological Resource Center Qilu Hospital of Shandong University Jinan Shandong China; ^3^ Research Institute of Breast Cancer Shandong University Jinan Shandong China

**Keywords:** anti‐tumor immunity, KDM3B, SHP1, STING, triple–negative breast cancer

## Abstract

Immunotherapy has emerged as a promising therapeutic option for cancer management, but its applicability in patients with triple–negative breast cancer (TNBC) is limited by the low efficacy due to the immunosuppressive tumor microenvironment (TME). Here, we identify lysine demethylase 3B (KDM3B) as an essential mediator of immune evasion in TNBC. KDM3B expression is negatively correlated with cytotoxic T lymphocyte (CTL) infiltration. Genetic or pharmacologic inhibition of KDM3B facilitates the recruitment and activation of CD8^+^ T cells, thereby suppressing tumor growth in TNBC mouse models. Mechanistically, KDM3B targets SHP1 by reducing H3K9me2 levels at its promoter. Suppression of KDM3B attenuates SHP1–mediated STING inactivation, which triggers robust type I interferon (IFN) responses. Strikingly, both KDM3B depletion and treatment with the KDM3B–selective inhibitor P3FI–90 significantly suppresses tumor progression and mitigates resistance to immune checkpoint blockade (ICB) therapy. Taken together, these findings establish KDM3B as a key regulator of immune escape, and targeting KDM3B represents a promising strategy to augment the efficacy of immunotherapy for TNBC.

## Graphical abstract

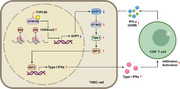

**Abbreviations**
ATAC–seq: assay for transposase–accessible chromatin with high throughput sequencing
ATCC: American Type Culture Collection
BCIP: Breast Cancer Integrative Platform
cGAMP: cyclic GMP–AMP
cGAS: cyclic GMP–AMP synthase
ChIP: chromatin immunoprecipitation
co–IP: co–immunoprecipitation
CRC: colorectal cancer
CTL: cytotoxic T lymphocyte
DAVID: Database for Annotation, Visualization and Integrated Discovery
DEG: differentially expressed gene
dsDNA: double–stranded DNA
ER: estrogen receptor
ERV: endogenous retrovirus
ESCC: esophageal squamous cell carcinoma
FBS: fetal bovine serum
GO: gene ontology
GSEA: gene set enrichment analysis
GZMB: granzyme B
HER2: human epidermal growth factor receptor 2
ICB: immune checkpoint blockade
ICD: immunogenic cell death
ICI: immune checkpoint inhibitor
IFN: interferon
IHC: immunohistochemistry
IRF3: interferon–regulatory factor 3
ISG: interferon–stimulated gene
KDM3B: lysine demethylase 3B
KEGG: Kyoto Encyclopedia of Genes and Genomes
METABRIC: Molecular Taxonomy of Breast Cancer International Consortium
MTT: methylthiazolyldiphenyl–tetrazolium bromide
pCR: pathological complete response
PD–L1: programmed death ligand–1
PR: progesterone receptor
RNA–seq: RNA–sequencing
RT–qPCR: real–time quantitative PCR
SHP1: SH2 domain–containing phosphatase 1
siRNA: small interfering RNA
STING: stimulator of interferon genes
STR: short tandem repeat
TBK1: TANK–binding protein 1
TCGA: The Cancer Genome Atlas
TIDE: Tumor Immune Dysfunction and Exclusion
TIL: tumor–infiltrating lymphocyte
TMB: tumor mutation burden
TME: tumor microenvironment
TNBC: triple–negative breast cancer
WT: wild–type

## 1 Introduction

According to the latest data from the American Cancer Society, breast cancer remains the most prevalent malignancy and the second leading cause of cancer–related mortality among women [1]. Triple–negative breast cancer (TNBC) accounts for 15%–20% of all breast cancers and is characterized by the absence of estrogen receptor (ER), progesterone receptor (PR), and human epidermal growth factor receptor 2 (HER2) expression [2]. TNBC is the most aggressive breast cancer subtype, characterized by poor prognosis, frequent recurrence, and high metastatic potential [3]. Despite recent therapeutic advances, metastatic and treatment–resistant TNBC continues to pose significant clinical challenges. Recently, immunotherapy, particularly immune checkpoint blockade (ICB) therapy, has revolutionized the treatment landscape of TNBC [4, 5]. Although TNBC exhibits features such as high tumor mutation burden (TMB) and programmed death ligand–1 (PD–L1) expression that may favor ICB responsiveness, clinical benefits remain limited due to low response rates [6, 7, 8]. The principal reason is that the immunosuppressive tumor microenvironment (TME) impedes CD8^+^ T cell infiltration and function, thereby impairing anti‐tumor immune responses. Therefore, novel strategies to remodel the TME and potentiate CD8^+^ T cell–mediated anti‐tumor immunity in TNBC are urgently needed.

Stimulator of interferon genes (STING) is regarded as a primary mediator of cytolytic CD8^+^ T cell activation and a critical therapeutic target for cancer immunotherapy [9, 10]. Upon recognizing double–stranded DNA (dsDNA), cyclic GMP–AMP synthase (cGAS) synthesizes and releases cyclic GMP–AMPs (cGAMPs), which activate STING [11]. Once activated, STING recruits TANK–binding protein 1 (TBK1), leading to phosphorylation, dimerization, and nuclear translocation of interferon–regulatory factor 3 (IRF3), thereby inducing the transcription of type I interferons (IFNs) and interferon–stimulated genes (ISGs) [12, 13]. Type I IFNs release and ISG induction drive robust anti‐tumor responses, including the recruitment of cytotoxic CD8^+^ T cells [14]. Recent studies have highlighted the regulation of the STING pathway in cancer cells. In esophageal squamous cell carcinoma (ESCC), HMGA1 represses *STING* transcription, thereby hindering the infiltration of tumor–infiltrating lymphocytes (TILs) and promoting ESCC progression [15]. In cervical cancer, BAG2 stabilizes STING by restricting the STUB1–mediated ubiquitination and degradation of STING, which inhibits the growth and metastasis of cervical cancer [16]. Although the molecular mechanisms of STING in various cancers have been well elucidated, how epigenetic regulators participate in modulating STING signaling, specifically in TNBC, remains largely unknown.

As a member of the jumonji C protein family, lysine demethylase 3B (KDM3B) regulates transcription by eliminating mono– and di–methylation of histone H3 specifically at lysine 9 (H3K9me1/2) [17]. Studies have shown that KDM3B is involved in diverse biological activities, including spermatogenesis, somatic growth, cell development, and cancer progression [18]. The tumor–promoting effects of KDM3B have been elucidated in various cancers. In colorectal cancer (CRC), KDM3B promotes tumor growth and chemoresistance by directly binding to promoter regions and activating the transcription of Wnt target genes [19]. In another study, a reduction in KDM3B expression was shown to have an antiproliferative effect on castration resistant prostate cancer [20]. Moreover, Khan et al. developed the KDM3B–selective inhibitor P3FI–90 and reported that P3FI–90 treatment significantly suppressed the growth and metastasis of fusion–positive rhabdomyosarcoma [21]. These findings indicate that KDM3B may serve as a therapeutic target. However, the functional role of KDM3B in TNBC and whether KDM3B contributes to cancer immunity have not been investigated.

In this study, we demonstrate that KDM3B functions as an immunosuppressor that restrains anti‐tumor immunity in TNBC. The inhibition of KDM3B activates the STING–type I IFN signaling pathway by increasing H3K9me2 modification at the promoter region of SH2 domain–containing phosphatase 1 (SHP1). Additionally, suppression of KDM3B significantly mitigates resistance to anti–PD–1 therapy. Overall, our findings highlight the potential of KDM3B as a therapeutic target for improving the clinical outcomes of immunotherapy for TNBC.

## 2 Results

### 2.1 Elevated KDM3B Expression is Correlated with Poor Overall Survival and Immunosuppression in TNBC

**FIGURE 1:**
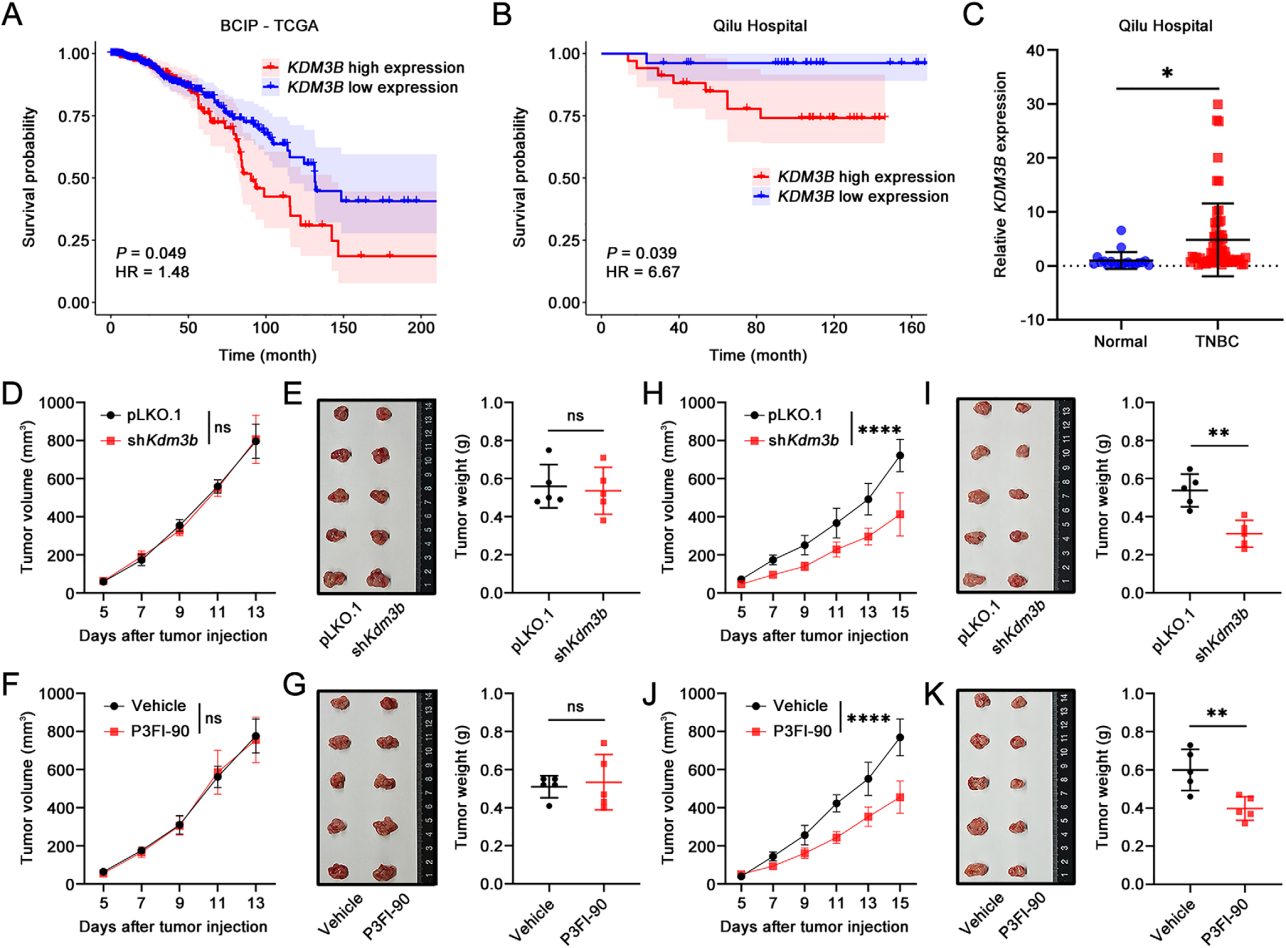
KDM3B is overexpressed in TNBC and correlated with immunosuppression. (A, B) Kaplan–Meier survival curves comparing the high– and low–*KDM3B* expression groups in the TCGA cohort (high expression, *n* = 370; low expression, *n* = 650) using the Breast Cancer Integrative Platform (BCIP) website (http://omicsnet.org/bcancer/database/) (A) and in the Qilu Hospital cohort (high expression, *n* = 34; low expression, *n* = 26) (B). HR, Hazard Ratio. (C) mRNA expression levels of *KDM3B* in adjacent normal and TNBC tissues detected by RT–qPCR (Normal, *n* = 19; TNBC, *n* = 60). (D, E) Tumor growth curves, tumor images, and tumor weight in immunodeficient nude mice subcutaneously injected with pLKO.1 or sh*Kdm3b* EMT6 cells (D, growth curves; E, tumor images and tumor weight). (F, G) Tumor growth curves, tumor images, and tumor weight in immunodeficient nude mice subcutaneously injected with EMT6 cells treated with vehicle or P3FI–90 (F, growth curves; G, tumor images and tumor weight). (H, I) Tumor growth curves, tumor images, and tumor weight in immunocompetent BALB/c mice subcutaneously injected with pLKO.1 or sh*Kdm3b* EMT6 cells (H, growth curves; I, tumor images and tumor weight). (J, K) Tumor growth curves, tumor images, and tumor weight in immunocompetent BALB/c mice subcutaneously injected with EMT6 cells treated with vehicle or P3FI–90 (J, growth curves; K, tumor images and tumor weight). *n* = 5 mice per group. Data are presented as means ± SD. Kaplan–Meier method in (A, B). Unpaired two–tailed Student's *t*–test in (C, E, G, I, K). Two–way ANOVA in (D, F, H, J). ns, not significant; ^*^ *p* < 0.05, ^**^ *p* < 0.01, ^****^ *p* < 0.0001.

To evaluate the association between KDM3B and breast cancer, we utilized the Breast Cancer Integrative Platform (BCIP) database and found that *KDM3B* expression was positively associated with poor prognosis (Figure 1A). We next assessed the role of *KDM3B* in TNBC and found that high *KDM3B* expression was associated with short overall survival in the Qilu Hospital cohort (Figure 1B). Furthermore, compared with adjacent normal tissues, TNBC tissues showed increased *KDM3B* mRNA levels (Figure 1C). Thus, KDM3B likely functions as an oncogene in TNBC.

We next investigated the functional role of KDM3B inhibition in vitro. We used sh*Kdm3b* plasmids to stably knock down the expression of *Kdm3b* and the highly selective KDM3B inhibitor P3FI–90 to suppress its demethylase activity in 4T1 and EMT6 murine TNBC cell lines (Figure S1A,B). However, neither genetic inhibition of *Kdm3b* nor pharmacological inhibition of KDM3B affected the proliferation or colony formation of tumor cells (Figure S1C–F). EMT6 cells were then subcutaneously injected into immunodeficient nude mice for in vivo studies. Neither *Kdm3b* knockdown nor inhibition of KDM3B demethylase activity affected tumor growth (Figure 1D–G). In contrast, tumors in the KDM3B inhibition groups showed reduced size and weight compared with the control groups in immunocompetent mice (Figure 1H–K). These results suggest that KDM3B suppression represses tumor growth in an immune–dependent manner.

### 2.2 Inhibition of KDM3B Potentiates CD8^+^ T Cell–Mediated Anti‐tumor Immunity

**FIGURE 2:**
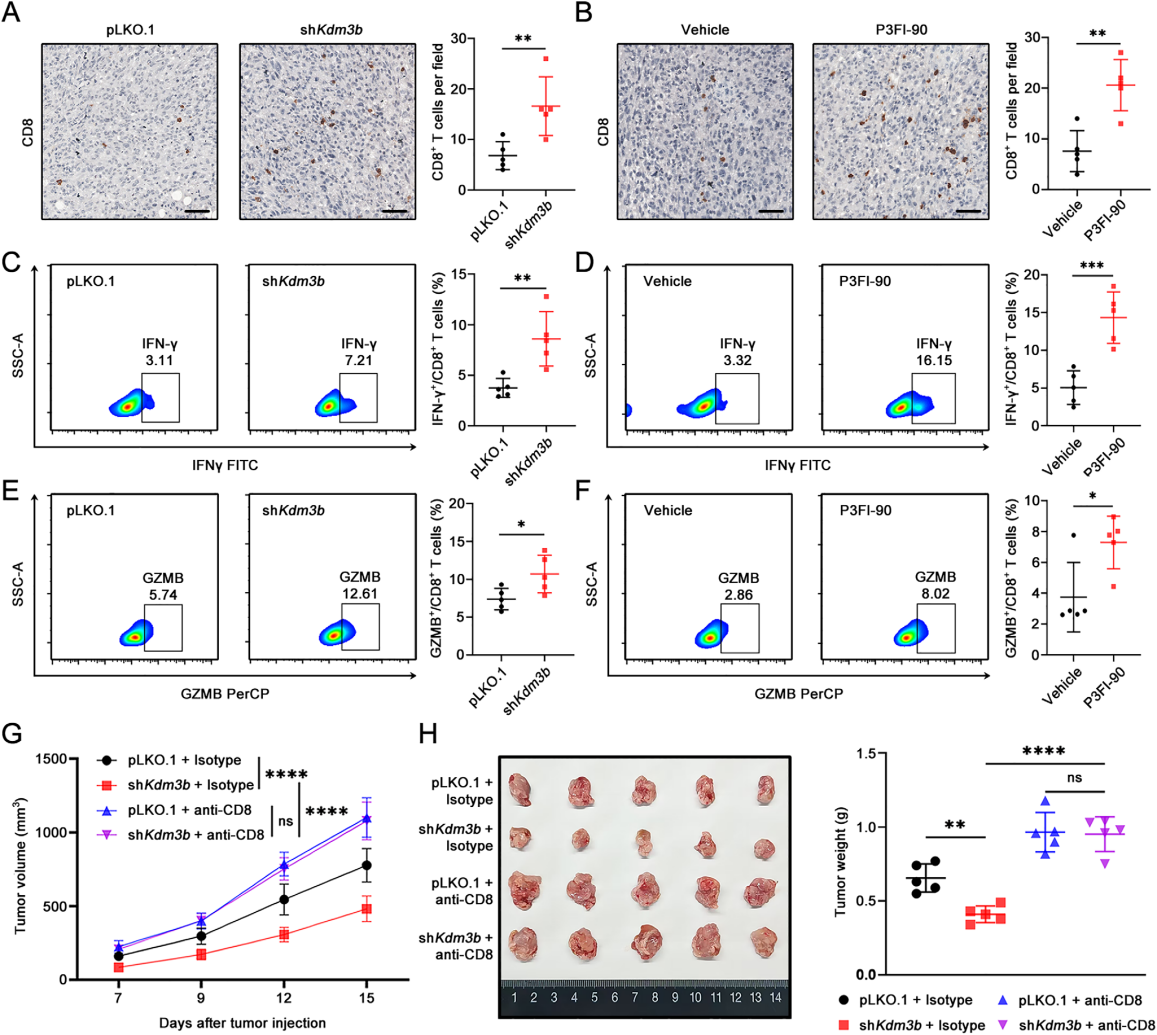
Inhibition of KDM3B promotes CD8^+^ T cell infiltration and activation. (A, B) IHC staining of CD8^+^ T cell infiltration in EMT6 xenografts treated with KDM3B inhibition (sh*Kdm3b* or P3FI–90). Scale bar, 50 µm. (C, D) Quantification of IFN–γ^+^CD8^+^ T cells in EMT6 xenografts treated with KDM3B inhibition (sh*Kdm3b* or P3FI–90). (E, F) Quantification of GZMB^+^CD8^+^ T cells in EMT6 xenografts treated with KDM3B inhibition (sh*Kdm3b* or P3FI–90). (G, H) Tumor growth curves, tumor images, and tumor weight of pLKO.1 or sh*Kdm3b* EMT6 xenografts treated with control IgG or anti–CD8α antibody (G, growth curves; H, tumor images and tumor weight). *n* = 5 mice per group. Data are presented as means ± SD. Unpaired two–tailed Student's *t*–test in (A–F, H). Two–way ANOVA in (G). ns, not significant; ^*^ *p* < 0.05, ^**^ *p* < 0.01, ^***^ *p* < 0.001, ^****^ *p* < 0.0001.

To explore the relationship between KDM3B and anti‐tumor immunity, we performed immunohistochemistry (IHC) assays and observed increased recruitment of CD8^+^ T cells in sh*Kdm3b* and P3FI–90 treated tumors (Figure 2A,B). Additionally, the results of flow cytometry assays revealed that *Kdm3b* knockdown and P3FI–90 treatment facilitated the infiltration of CD3^+^ T cells and CD8^+^ T cells, but not CD45^+^ cells (Figures S2 and S3A–F). KDM3B inhibition also increased the synthesis of IFN–γ and granzyme B (GZMB) by CD8^+^ T cells (Figure 2C–F).

To determine whether KDM3B suppression–mediated anti‐tumor immunity is dependent on CD8^+^ T cells, we depleted CD8^+^ T cells using a CD8α neutralizing antibody. We found that CD8^+^ T cell depletion markedly eliminated the differences in tumor burden between the pLKO.1 and sh*Kdm3b* groups (Figure 2G, H, and Figure S3G). These results indicate that inhibition of KDM3B enhances CD8^+^ T cell–mediated anti‐tumor immunity.

### 2.3 Inhibition of KDM3B Induces type I IFN Responses in Tumor Cells

**FIGURE 3:**
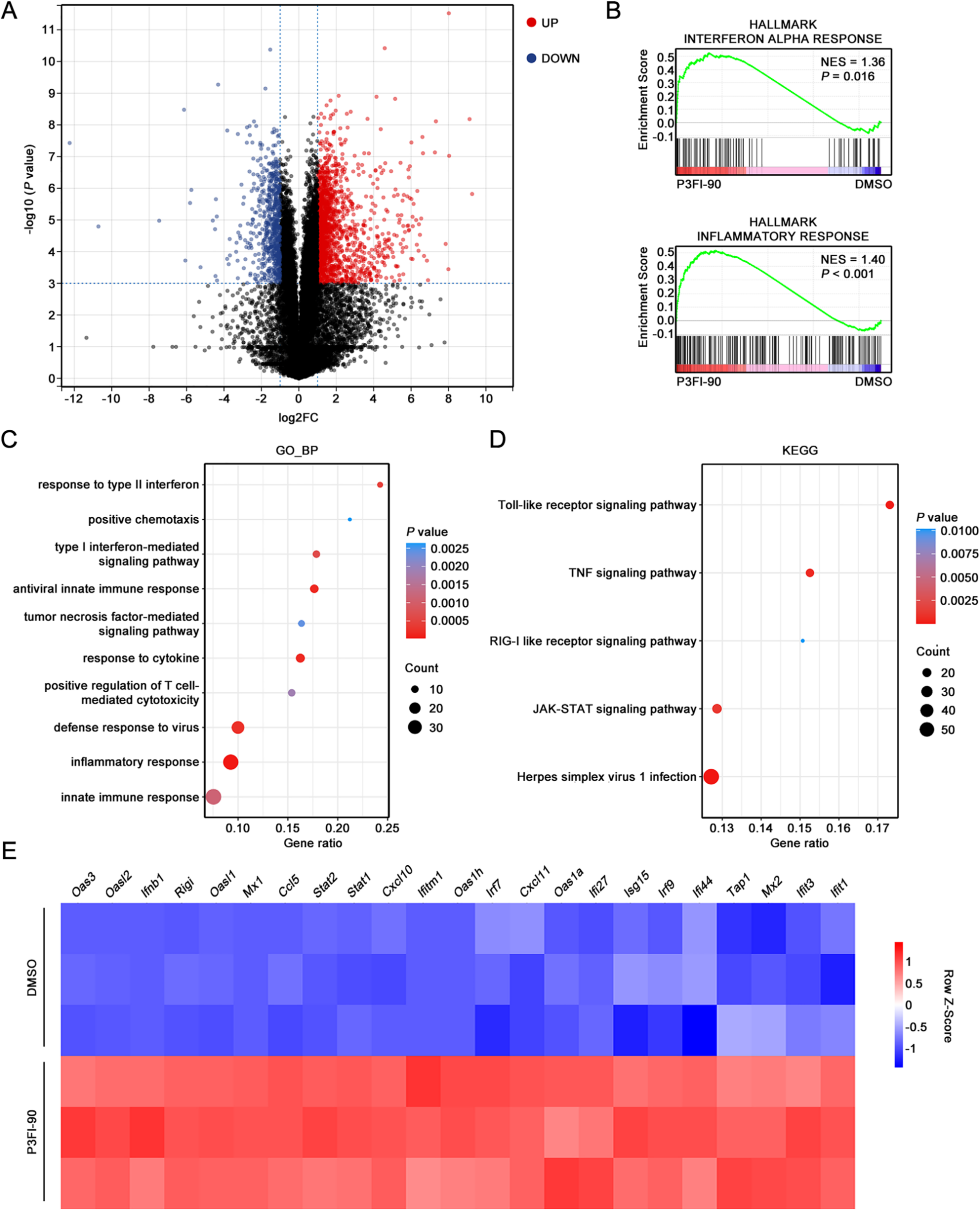
Inhibition of KDM3B is related to innate immune responses. (A) Volcano plot showing differentially expressed genes (DEGs) between DMSO– and P3FI–90–treated cells. (B) Gene set enrichment analysis (GSEA) identified two hallmark signatures induced by P3FI–90 treatment. NES, non–normalized enrichment score. (C, D) Gene ontology (GO) and Kyoto encyclopedia of genes and genomes (KEGG) analyses identified innate immune response–related pathways induced by P3FI–90 treatment. (E) Heatmap of DEGs between DMSO– and P3FI–90–treated cells.

To elucidate how KDM3B suppression enhances CD8^+^ T cell recruitment and function, we performed transcriptomic RNA–sequencing (RNA–seq) (GSE330140) on DMSO– and P3FI–90–treated 4T1 cells. A volcano plot shows differentially expressed genes (DEGs) between the DMSO and P3FI–90 groups (Figure 3A). Furthermore, gene set enrichment analysis (GSEA) revealed that pathways associated with type I IFN signaling were highly enriched in P3FI–90–treated cells (Figure 3B). Additionally, P3FI–90 treatment activated innate immune pathways according to gene ontology (GO) and Kyoto Encyclopedia of Genes and Genomes (KEGG) analyses (Figure 3C,D). The heatmap shows that a subset of ISGs was upregulated by P3FI–90 treatment (Figure 3E). GO, KEGG, and GSEA analyses derived from The Cancer Genome Atlas (TCGA) and Molecular Taxonomy of Breast Cancer International Consortium (METABRIC) databases revealed that reduced *KDM3B* expression was closely associated with the innate immune response and type I IFN activation (Figure S4A–E). Furthermore, *KDM3B* mRNA levels were negatively correlated with several ISGs and T cell markers (Figure S4F,G). In addition, *KDM3B* expression was negatively correlated with cytotoxic T lymphocyte (CTL) infiltration according to the Tumor Immune Dysfunction and Exclusion (TIDE) website (Figure S5). These results indicate that KDM3B inhibition activates type I IFN signaling.

**FIGURE 4:**
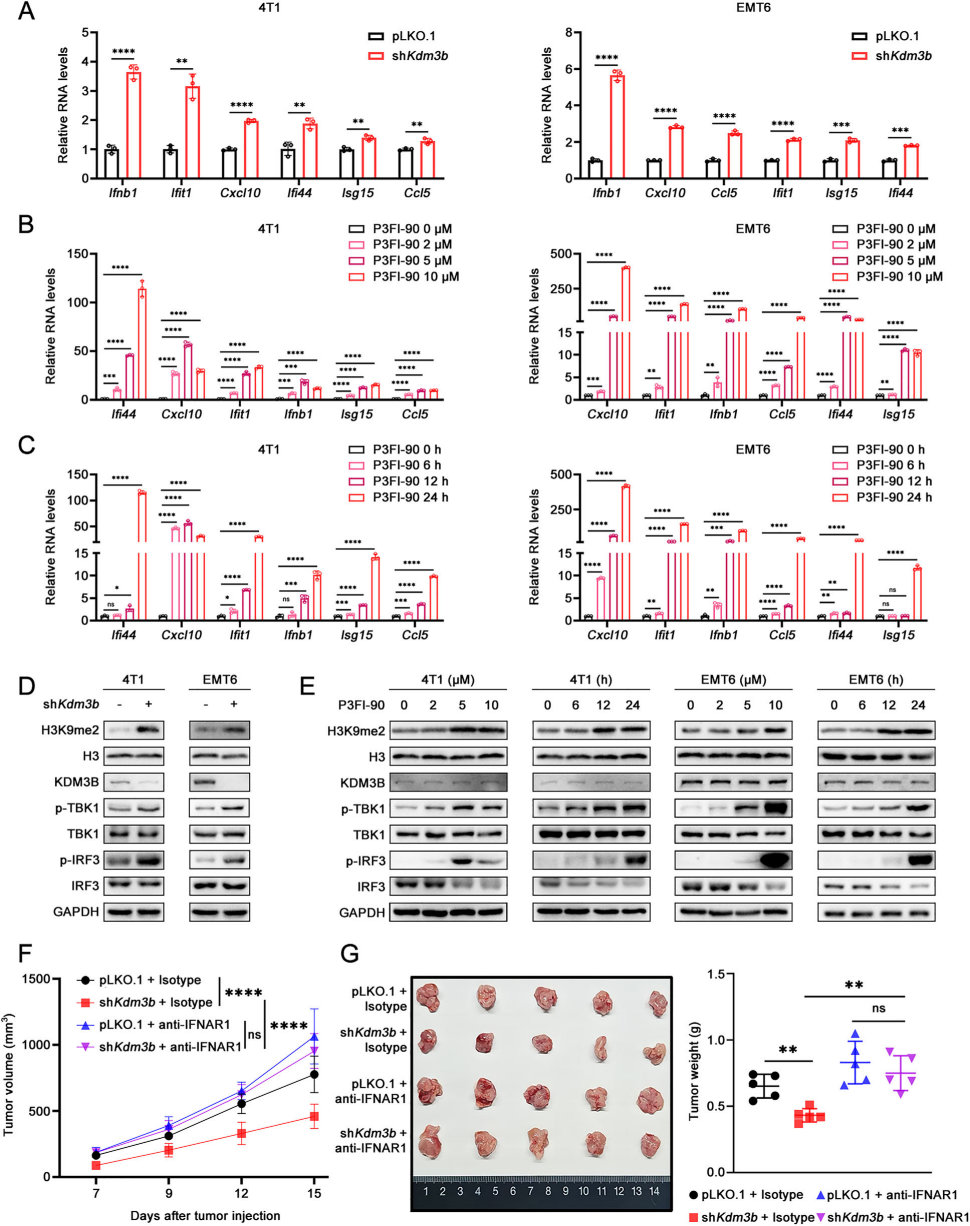
KDM3B inhibition–mediated anti‐tumor immunity is dependent on type I IFN. (A) Expression of *Ifnb1* and interferon–stimulated genes (ISGs) measured by RT–qPCR following *Kdm3b* knockdown in TNBC cells. (B) Expression of *Ifnb1* and ISGs measured by RT–qPCR in TNBC cells treated with increasing concentrations of P3FI–90 (24 h). (C) Expression of *Ifnb1* and ISGs measured by RT–qPCR in TNBC cells treated with P3FI–90 (10 µm) over time. (D) Activation of TBK1 and IRF3 was detected by Western blotting following *Kdm3b* knockdown in TNBC cells. (E) Activation of TBK1 and IRF3 detected by Western blotting following P3FI–90 treatment in TNBC cells. (F, G) Tumor growth curves, tumor images, and tumor weight of pLKO.1 or sh*Kdm3b* EMT6 xenografts treated with control IgG or anti–IFNAR1 antibody (F, growth curves; G, tumor images and tumor weight). *n* = 5 mice per group. Data are presented as means ± SD. Unpaired two–tailed Student's *t*–test in (A–C, G). Two–way ANOVA in (F). ns, not significant; ^*^ *p* < 0.05, ^**^ *p* < 0.01, ^***^ *p* < 0.001, ^****^ *p* < 0.0001.

To further investigate whether KDM3B suppression could induce robust type I IFN responses, real–time quantitative PCR (RT–qPCR) assays were performed to detect changes in the mRNA levels of multiple ISGs. We observed that KDM3B inhibition increased the mRNA expression of *Ifnb1* and ISGs, including *Ccl5*, *Cxcl10*, *Isg15*, *Ifi44*, and *Ifit1* (Figure 4A–C). The TBK1–IRF3 axis is important for the generation of type I IFNs. As validated by Western blot assays, *Kdm3b* knockdown and P3FI–90 treatment promoted the phosphorylation of TBK1 and IRF3 at the protein level (Figure 4D, E).

Considering the vital role of type I IFNs in anti‐tumor immune responses, we speculated that targeting KDM3B restrains tumor growth via type I IFNs. To validate our hypothesis, we applied an IFNAR1 neutralizing antibody to eliminate type I IFN responses in tumors. Consequently, blockade of type I IFNs markedly reversed the reduced tumor growth caused by *Kdm3b* knockdown (Figure 4F,G). These results suggest that type I IFNs are indispensable for the anti‐tumor effects of KDM3B inhibition.

### 2.4 Inhibition of KDM3B Promotes STING Activation Independent of cGAS

**FIGURE 5:**
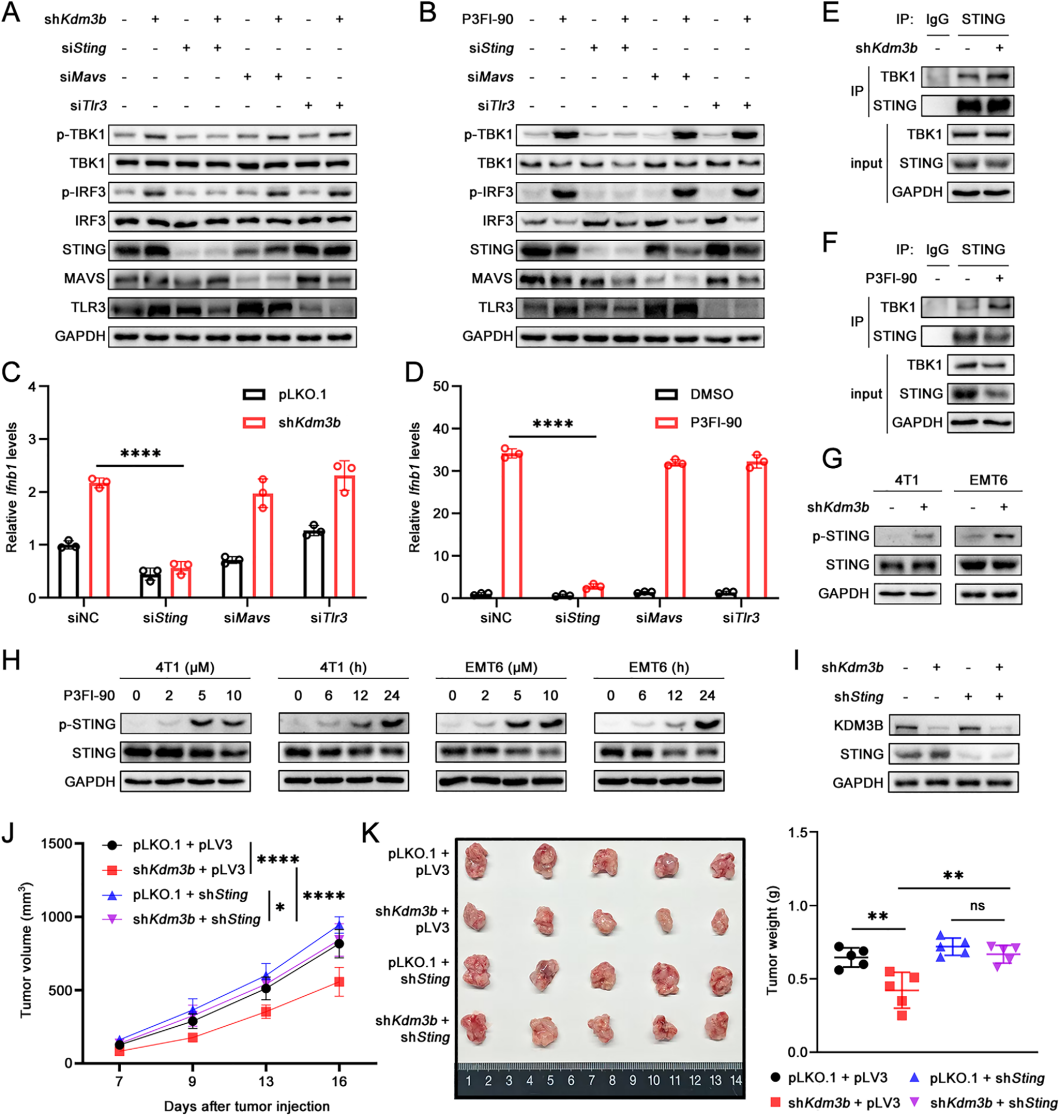
Inhibition of KDM3B activates the STING–type I IFN pathway. (A, B) Effects of KDM3B inhibition (sh*Kdm3b* or P3FI–90, 10 µm, 24 h) or knockdown of intracellular sensors (*Sting*, *Mavs*, and *Tlr3*) on TBK1/IRF3 activation detected by Western blotting in EMT6 cells. (C, D) Effects of KDM3B inhibition (sh*Kdm3b* or P3FI–90, 10 µm, 24 h) or knockdown of cellular sensors (*Sting*, *Mavs*, and *Tlr3*) on *Ifnb1* mRNA expression levels detected by RT–qPCR in EMT6 cells. (E, F) Co–IP assays for assessing the STING–TBK1 interaction following KDM3B inhibition (sh*Kdm3b* or P3FI–90, 10 µm, 24 h) in EMT6 cells. (G, H) STING activation detected by Western blotting following KDM3B inhibition (sh*Kdm3b* or P3FI–90, 10 µm, 24 h) in TNBC cells. (I) Effects of sh*Kdm3b* and sh*Sting* on KDM3B and STING protein levels detected by Western blotting in EMT6 cells. (J, K) Tumor growth curves, tumor images, and tumor weight of EMT6 xenografts treated with sh*Kdm3b* or sh*Sting* (J, growth curves; K, tumor images and tumor weight). *n* = 5 mice per group. Data are presented as means ± SD. Unpaired two–tailed Student's *t*–test in (C, D, K). Two–way ANOVA in (J). ns, not significant; ^*^ *p* < 0.05, ^**^ *p* < 0.01, ^****^ *p* < 0.0001.

In general, the tumor–intrinsic TBK1–IRF3–type I IFN axis is triggered by distinct dsDNA or dsRNA sensor–mediated signaling, including cGAS–STING, RIG–I/MDA5–MAVS, and TLR3 signaling [22]. To determine which sensor contributes to KDM3B inhibition–mediated IFN–β production, we used small interfering RNAs (siRNAs) to disrupt the expression of *Sting*, *Mavs*, and *Tlr3*. Rescue experiments revealed that *Sting* knockdown, but not *Mavs* or *Tlr3*, reversed the activation of TBK1 and IRF3, as well as the transcription of *Ifnb1* (Figure 5A–D). Next, we investigated whether KDM3B inhibition affects the activation of STING. As shown in Figure 5E,F, KDM3B suppression promoted STING–TBK1 complex formation. Moreover, *Kdm3b* knockdown and P3FI–90 treatment promoted the phosphorylation of STING at Ser365. (Figure 5G,H). These results indicate that inhibition of KDM3B stimulates type I IFN responses through the activation of STING.

We next aimed to elucidate the role of STING in KDM3B suppression–regulated tumor growth inhibition. We stably knocked down *Sting* expression in control and sh*Kdm3b* EMT6 cells (Figure 5I). As expected, *Sting* deficiency significantly abrogated the tumor growth regression caused by sh*Kdm3b* (Figure 5J,K). Additionally, *Sting* knockdown reversed P3FI–90–induced tumor growth inhibition, and enhanced CD8^+^ T cell infiltration and activation (Figure S6A–E). Collectively, these data imply that the anti‐tumor effects of KDM3B suppression are mediated through tumor–intrinsic STING signaling.

To investigate whether cGAS participates in the STING activation induced by KDM3B repression, we used a specific siRNA against *Cgas*. However, *Cgas* knockdown did not prevent the phosphorylation of STING, TBK1, or IRF3 induced by KDM3B inhibition or the increase in *Ifnb1* mRNA expression (Figure S7A–D). The activation of STING, TBK1, and IRF3 was completely reversed by the STING inhibitor H–151, but not by the cGAS inhibitor RU.521 (Figure S7E,F). In addition, compared with RU.521 treatment, H–151 treatment significantly increased the inhibitory effect on *Ifnb1* transcription (Figure S7G,H). Therefore, cGAS is not involved in KDM3B suppression–mediated STING activation.

### 2.5 KDM3B Regulates SHP1 Expression via H3K9me2

**FIGURE 6:**
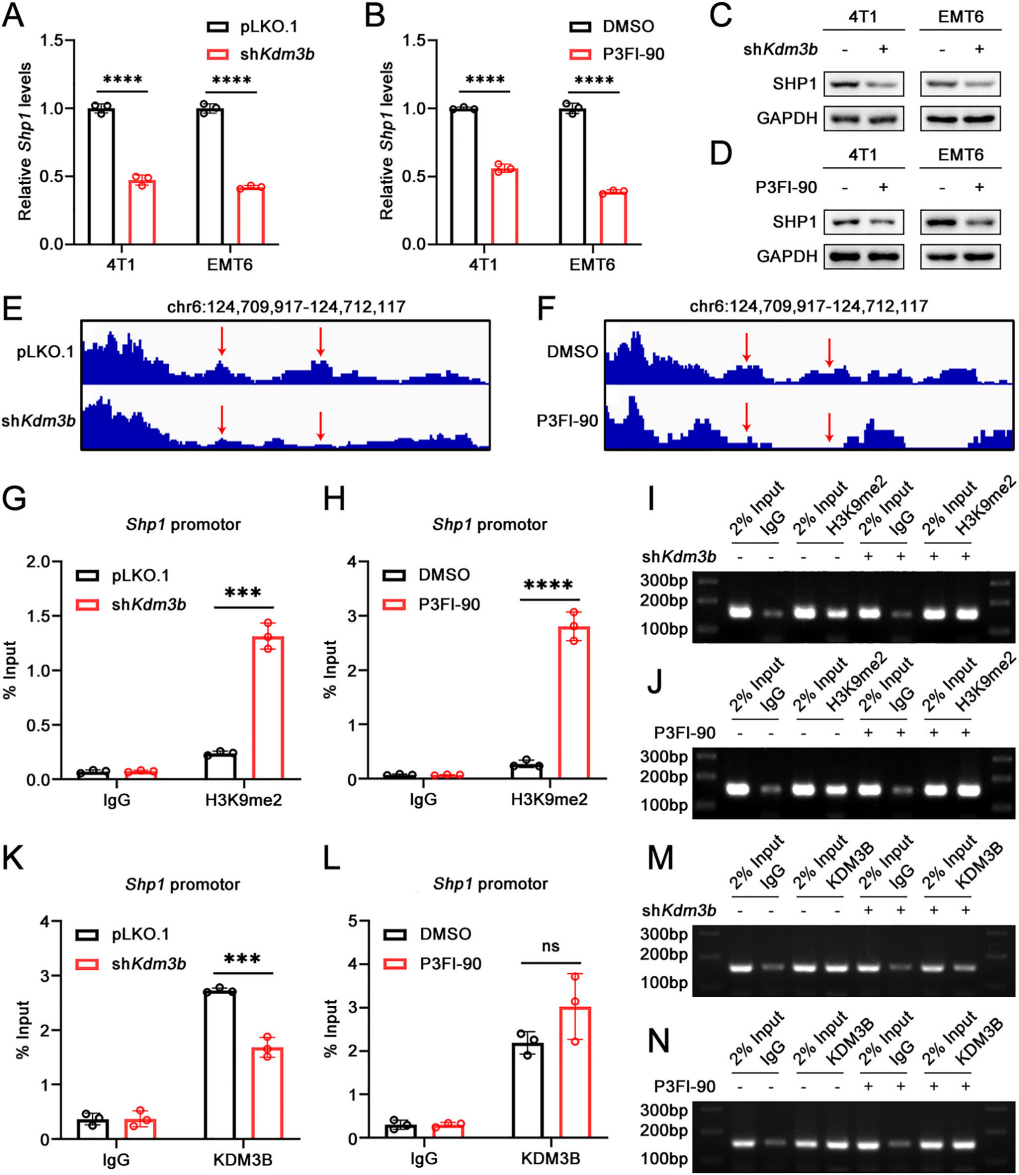
Inhibition of KDM3B suppresses SHP1 expression by increasing H3K9me2 at the *Shp1* promoter. (A, B) Expression of *Shp1* measured by RT–qPCR following KDM3B inhibition (sh*Kdm3b* or P3FI–90, 10 µm, 24 h) in TNBC cells. (C, D) SHP1 protein expression detected by Western blotting following KDM3B inhibition (sh*Kdm3b* or P3FI–90, 10 µm, 24 h) in TNBC cells. (E, F) Integrative Genomic Viewer (IGV) screenshots of aggregated ATAC–seq signals at the *Shp1* promoter. (G, H) ChIP–qPCR assays assessing H3K9me2 enrichment at the *Shp1* promoter following KDM3B inhibition (sh*Kdm3b* or P3FI–90, 10 µm, 24 h) in EMT6 cells. (I, J) Representative gel electrophoresis result images. (K, L) ChIP–qPCR assays assessing KDM3B binding at the *Shp1* promoter following KDM3B inhibition (sh*Kdm3b* or P3FI–90, 10 µm, 24 h) in EMT6 cells. (M, N) Representative gel electrophoresis result images. Data are presented as means ± SD. Unpaired two–tailed Student's *t*–test in (A, B, G, H, K, L). ns, not significant; ^***^ *p* < 0.001, ^****^ *p* < 0.0001.

KDM3B has been reported to erase H3K9me2 at the promoter of its target genes, thereby promoting transcription [18]. Given that KDM3B inhibition positively induces STING–dependent type I IFN responses, we focused on DEGs downregulated by P3FI–90 treatment. Through literature search, three genes that directly inactivate STING signaling were identified: *APEX1*, *DHCR24*, and *SHP1* [23, 24, 25]. A heatmap displays the differential expression of these genes between control and P3FI–90–treated 4T1 cells (Figure S8A). Next, on the basis of RT–qPCR assays, we observed that only *Shp1* mRNA expression decreased in sh*Kdm3b* or P3FI–90–treated TNBC cells (Figure 6A,B, and Figure S8B,C). Immunoblotting further confirmed that KDM3B suppression reduced SHP1 expression at the protein level (Figure 6C,D).

To elucidate the epigenetic mechanisms by which KDM3B regulates SHP1 expression, we performed an assay for transposase–accessible chromatin with high‐throughput sequencing (ATAC–seq) (GSE329777) to assess KDM3B–dependent alterations in the chromatin landscape. Notably, we observed that KDM3B inhibition significantly decreased chromatin accessibility at the *Shp1* promoter region, confirming the chromatin accessibility–regulating effect of KDM3B on the *Shp1* promoter (Figure 6E, F). We next assessed the changes in H3K9me2 levels and KDM3B binding at the *Shp1* promoter after KDM3B inhibition. Increased H3K9me2 levels at the *Shp1* promoter were detected after KDM3B inhibition by chromatin immunoprecipitation (ChIP)–PCR assays (Figure 6G–J). *Kdm3b* knockdown decreased, whereas P3FI–90 treatment had a minimal effect on the interaction between KDM3B and the *Shp1* promoter (Figure 6K–N), indicating that P3FI–90 treatment does not affect the DNA binding ability of KDM3B.

To confirm that KDM3B inhibition suppresses SHP1 expression by increasing H3K9me2 levels at the *Shp1* promoter, we overexpressed wild–type (WT) *Kdm3b* or *Kdm3b* with an H1561A mutation (expressing catalytically inactive KDM3B) in KDM3B–inhibited EMT6 cells. We found that the demethylase activity of KDM3B reversed the decrease in *Shp1* expression and increase in H3K9me2 levels at the *Shp1* promoter. These rescue experiments provide strong causal evidence that KDM3B regulates *Shp1* expression via H3K9me2 (Figure S9A–H). Moreover, P3FI–90–induced SHP1 downregulation was not rescued by *Kdm3a* or *Kdm4a–d* expression (Figure S10). These data suggest that P3FI–90 is specific for KDM3B in TNBC.

### 2.6 Inhibition of KDM3B Impairs SHP1–Mediated STING Inactivation

**FIGURE 7:**
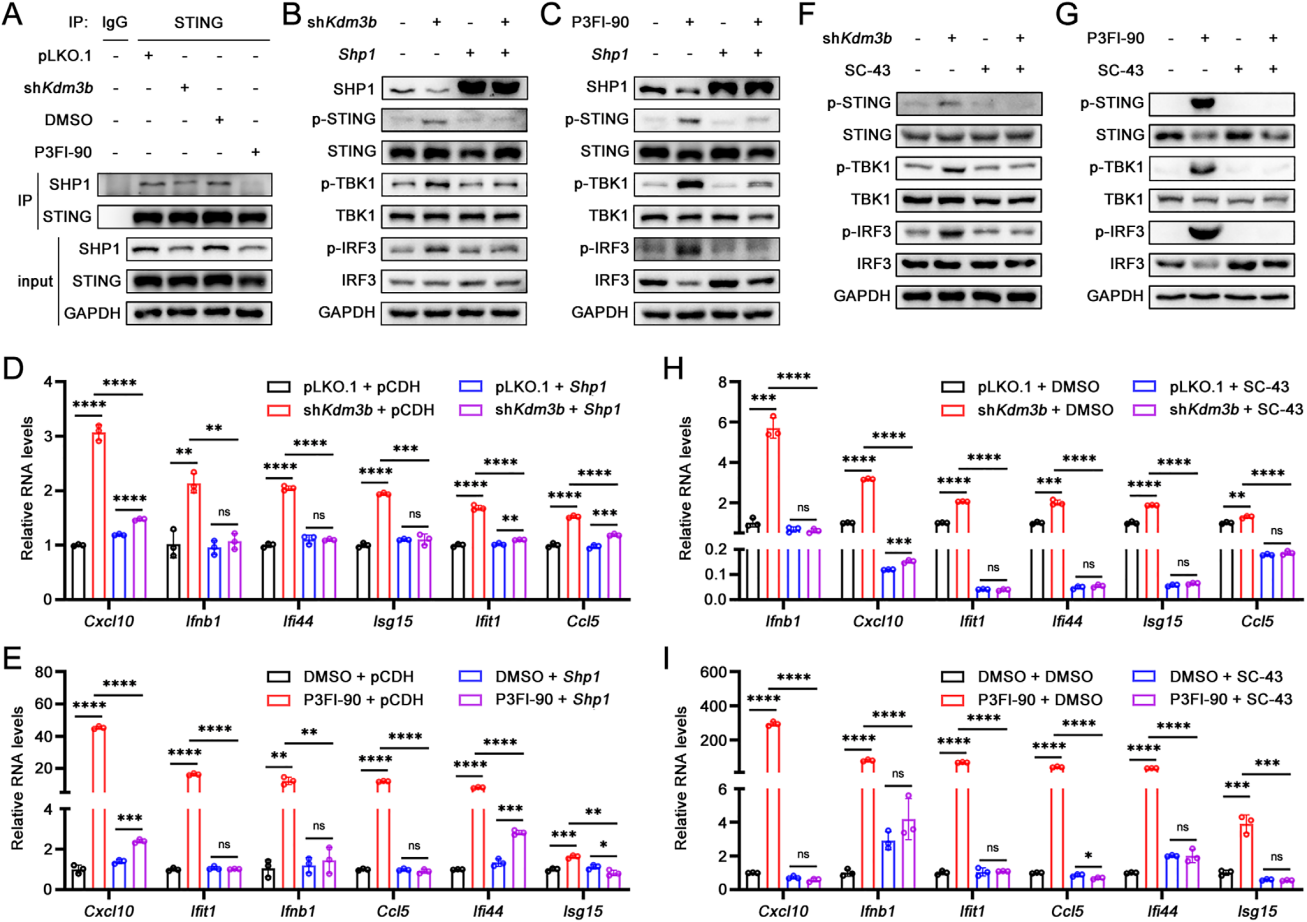
Inhibition of KDM3B triggers STING signaling by suppressing SHP1. (A) co–IP assays assessing the effects of KDM3B inhibition (sh*Kdm3b* or P3FI–90, 10 µm, 24 h) on the interaction between SHP1 and STING in EMT6 cells. (B, C) Effects of KDM3B inhibition (sh*Kdm3b* or P3FI–90, 10 µm, 24 h) or *Shp1* overexpression on STING, TBK1, and IRF3 activation detected by Western blotting in EMT6 cells. (D, E) Effects of KDM3B inhibition (sh*Kdm3b* or P3FI–90, 10 µm, 24 h) or *Shp1* overexpression on *Ifnb1* and ISGs mRNA expression levels detected by RT–qPCR in EMT6 cells. (F, G) Effects of KDM3B inhibition (sh*Kdm3b* or P3FI–90, 10 µm, 24 h) or SC–43 (10 µm, 24 h) on STING, TBK1, and IRF3 activation detected by Western blotting in EMT6 cells. (H, I) Effects of KDM3B inhibition (sh*Kdm3b* or P3FI–90, 10 µm, 24 h) or SC–43 (10 µm, 24 h) on *Ifnb1* and ISGs mRNA expression levels detected by RT–qPCR in EMT6 cells. Data are presented as means ± SD. Unpaired two–tailed Student's *t*–test in (D, E, H, I). ns, not significant; ^*^ *p* < 0.05, ^**^ *p* < 0.01, ^***^ *p* < 0.001, ^****^ *p* < 0.0001.

It has been reported that SHP1 inactivates STING by directly binding to STING [25, 26]. Therefore, we investigated whether KDM3B inhibition interferes with the SHP1–STING interaction. As shown in Figure 7A, KDM3B inhibition reduced the binding between SHP1 and STING. More importantly, compared with *Kdm3b* knockdown, P3FI–90 treatment exhibited a stronger inhibitory effect on the SHP1–STING interaction (Figure 7A).

To determine the role of SHP1 in the regulation of STING–TBK1–IRF3–IFNB1–ISG axis by KDM3B inhibition, we restored *Shp1* expression in sh*Kdm3b* or P3FI–90–treated cells. *Shp1* overexpression reversed the KDM3B inhibition–mediated activation of STING, TBK1, and IRF3, as well as the increase in mRNA expression of *Ifnb1* and ISGs (Figure 7B–E). Furthermore, the activation of type I IFN signaling was completely reversed by the SHP1 agonist SC–43 (Figure 7F–I). These findings suggest that inhibition of KDM3B activates the STING–type I IFN axis by downregulating SHP1.

### 2.7 Targeting KDM3B Enhances the Therapeutic Efficacy of ICB Therapy

**FIGURE 8:**
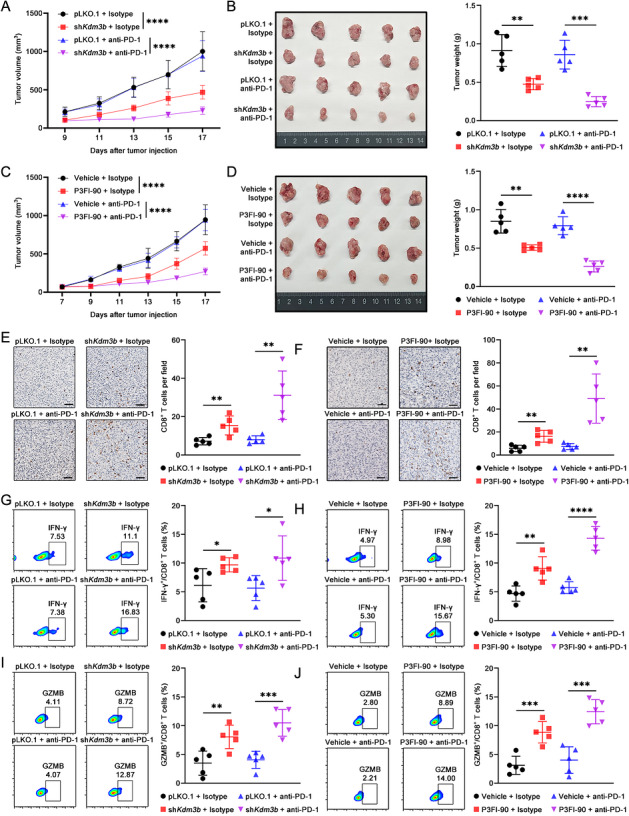
Targeting KDM3B enhances the response to anti–PD–1 therapy. (A, B) Tumor growth curves, tumor images, and tumor weight of pLKO.1 or sh*Kdm3b* EMT6 xenografts treated with control IgG or anti–PD–1 antibody (A, growth curves; B, tumor images and tumor weight). (C, D) Tumor growth curves, tumor images, and tumor weight of EMT6 xenografts treated with vehicle or P3FI–90 combined with control IgG or anti–PD–1 antibody (C, growth curves; D, tumor images and tumor weight). (E, F) IHC staining of CD8^+^ T cell infiltration in EMT6 xenografts treated with KDM3B inhibition (sh*Kdm3b* or P3FI–90) combined with control IgG or anti–PD–1 antibody. Scale bar, 50 µm. (G, H) Quantification of IFN–γ^+^CD8^+^ T cells in EMT6 xenografts treated with KDM3B inhibition (sh*Kdm3b* or P3FI–90) combined with control IgG or anti–PD–1 antibody. (I, J) Quantification of GZMB^+^CD8^+^ T cells in EMT6 xenografts treated with KDM3B inhibition (sh*Kdm3b* or P3FI–90) combined with control IgG or anti–PD–1 antibody. *n* = 5 mice per group. Data are presented as means ± SD. Unpaired two–tailed Student's *t*–test in (B, D–J). Two–way ANOVA in (A, C). ^*^ *p* < 0.05, ^**^ *p* < 0.01, ^***^ *p* < 0.001, ^****^ *p* < 0.0001.

Given that KDM3B suppression enhances anti‐tumor immunity in a CD8^+^ T cell–dependent manner, we hypothesized that targeting KDM3B could improve the efficacy of anti–PD–1 therapy. To test this hypothesis, we combined KDM3B inhibition with PD–1 blockade. We found that EMT6 tumors were resistant to anti–PD–1 monotherapy (Figure 8A–D), which is consistent with the findings of previous studies [27, 28]. Notably, compared with monotherapy, the combination of KDM3B inhibition and anti–PD–1 therapy significantly reduced tumor size and weight (Figure 8A–D). We subsequently harvested the tumor samples for further experiments. IHC and flow cytometry assays indicated that combination therapy markedly promoted CD8^+^ T cell recruitment to the tumor site (Figure 8E, F, and Figure S11A,B). Moreover, flow cytometry assays revealed that KDM3B inhibition combined with anti–PD–1 therapy significantly increased IFN–γ and GZMB expression by CD8^+^ T cells (Figure 8G–J). Using the TIDE website, we distinguished immunotherapy responders and non–responders in the TCGA cohort, and found that immunotherapy non–responders had higher *KDM3B* expression (Figure S12). Taken together, our results provide strong evidence that targeting KDM3B may represent a potential therapeutic strategy to improve the efficacy of ICB therapy in TNBC.

## 3 Discussion

TNBC is considered the most intractable subtype of breast cancer owing to its poor prognosis and the lack of effective treatment options. Adjuvant and neoadjuvant chemotherapy regimens remain the primary approach for TNBC treatment, whereas some patients with TNBC derive limited benefit from chemotherapy due to unavoidable chemoresistance [29]. Immunotherapy, especially ICB therapy, is important in the treatment of patients with TNBC. Atezolizumab (anti–PD–L1) is the first immune checkpoint inhibitor (ICI) approved for metastatic TNBC [30]. The IMpassion031 clinical trial indicates that atezolizumab in combination with nab–paclitaxel and anthracycline can improve pathological complete response (pCR) rates in patients with early–stage TNBC [31]. In addition, the addition of pembrolizumab (an anti–PD–1 antibody) is beneficial for TNBC patients who do not achieve a pCR according to the KEYNOTE–522 clinical trial [32]. However, the low response rate to ICB therapies limits their application in patients with TNBC. In this study, we revealed that inhibition of KDM3B could enhance the efficacy of ICB therapy. Both *Kdm3b* knockdown and P3FI–90 treatment significantly alleviated resistance to anti–PD–1 therapy in EMT6 tumor–bearing mouse models. Furthermore, the proportions of IFN–γ^+^CD8^+^ and GZMB^+^CD8^+^ T cells were higher in the combination groups than in the control groups. In summary, KDM3B may serve as a biomarker to predict the response to immunotherapy in TNBC, and the use of the KDM3B inhibitor P3FI–90 may provide insights for the design of more suitable treatment strategies for TNBC.

Numerous studies have highlighted the crucial role of CD8^+^ T cell–mediated anti‐tumor immunity in TNBC. Notably, the presence of TILs, particularly CD8^+^ T cells, is closely associated with the response to neoadjuvant chemotherapy and predicts a favorable prognosis in TNBC patients [33, 34, 35]. The density of CD8^+^ T cell infiltration is closely linked to the efficacy of ICB therapy [36]. However, the TIME of TNBC is highly heterogeneous, and a subset of patients with TNBC exhibit low or even no CD8^+^ T cell infiltration, which leads to immunotherapy failure. Our in vivo experiments revealed that KDM3B inhibition enhanced CD8^+^ T cell migration to the tumor site and increased the cytotoxicity of CD8^+^ T cells. These findings suggest that KDM3B may regulate CD8^+^ T cell distribution in TNBC. The interaction between chemokines and their receptors contributes to the recruitment of immune cells to the tumor site. Among these chemokines, CXCL10, a selective ligand of CXCR3, facilitates the migration, differentiation, and activation of CD8^+^ T cells [37, 38]. In TNBC, high levels of CXCL10 predict prolonged survival, and CXCL10 can be used as a biomarker for ICB therapy [39, 40]. We found that KDM3B inhibition induced robust type I IFN responses and promoted the transcription of *Cxcl10*, a canonical ISG. These findings reveal the close relationship between KDM3B and CD8^+^ T cell–mediated anti‐tumor immunity in TNBC. Nevertheless, further mechanistic studies are needed to gain a more comprehensive understanding of the impact of KDM3B on the TIME of TNBC.

Recently, STING has emerged as a key transducer in innate immune responses and anti‐tumor immunity. It has been reported that the downregulation of tumor–intrinsic STING activation can result in resistance to immune killing [41]. Furthermore, insufficient STING activation reduces the infiltration of CD3^+^CD8^+^ T cells into tumors by decreasing the expression of type I IFNs and their downstream chemokines CXCL9 and CXCL10 [41]. Therefore, identifying strategies to restore STING activation in cancer cells is highly important. In our study, we confirmed that KDM3B inhibition induced STING–dependent type I IFN responses that were independent of MAVS or TLR3. KDM3B inhibition facilitated the expression of ISGs, the phosphorylation of STING, and the interaction between STING and TBK1. Studies have demonstrated the function of SHP1 in inactivating STING during viral infection and in cancer immunity [25, 26]. On the basis of our RNA–seq and ChIP–qPCR data, we identified SHP1 as a key negative regulator of KDM3B inhibition–mediated STING activation. The inhibition of KDM3B significantly disrupted the SHP1–STING interaction. Restoration of SHP1 expression and treatment with the SHP1 agonist SC–43 reversed the KDM3B inhibition–induced expression of ISGs, as well as the activation of STING, TBK1, and IRF3. Notably, we identified this non–canonical STING activation in a cGAS–independent manner. Knockdown of *Cgas* expression or treatment with RU.521, a cGAS inhibitor, had limited effects on KDM3B inhibition–mediated type I IFN responses. Thus, KDM3B may represent a novel molecular target to overcome the resistance to ICB caused by insufficient STING activation.

An increasing number of studies have focused on the impact of the KDM family on TIME remodeling. For instance, inhibition of the KDM4 family by JIB–04 modulates innate immune responses and immunogenic cell death (ICD), thereby repressing tumor growth [42]. In gastric cancer, deficiency of KDM3A triggers endogenous retrovirus (ERV)–induced IFN responses by regulating H3K4me2 [43]. Knockdown of KDM4C or treatment with SD70, a specific KDM4C inhibitor, promotes CXCL10–mediated CD8^+^ T cell recruitment and function by increasing H3K36me3 modification at the *Cxcl10* promoter in lung cancer. However, no study has investigated the functional role of KDM3B in TNBC, particularly its effects on cancer immunity. Although Paolicchi et al. reported that high KDM3B expression might predict a favorable prognosis in patients with breast cancer, our study demonstrates that KDM3B plays a tumor–promoting role in TNBC. Using qPCR assays, we detected higher *KDM3B* expression in TNBC tissues than in adjacent normal tissues. In vivo experiments revealed that KDM3B suppression restricted EMT6 tumor growth. Mechanistically, inhibition of KDM3B triggers STING–mediated anti‐tumor immunity by increasing H3K9me2 methylation at the *Shp1* promoter. More importantly, we evaluated the therapeutic potential of the KDM3B–selective inhibitor P3FI–90 for TNBC treatment. Kim et al. reported that P3FI–90 has the highest specificity for KDM3B. P3FI–90 significantly elevates H3K9me2 levels at the enhancer regions of *PAX3–FOXO1*, thereby repressing PAX3–FOXO1 function and promoting apoptosis in fusion–positive rhabdomyosarcoma [21]. Here, we demonstrated that in TNBC, P3FI–90 treatment had minimal effect on cell proliferation, whereas it boosted anti‐tumor immunity to suppress tumor growth. Additionally, P3FI–90 significantly mitigated resistance to anti–PD–1 therapy in the EMT6 tumor model, which provides potential therapeutic options for the treatment of TNBC in the future.

**FIGURE 9:**
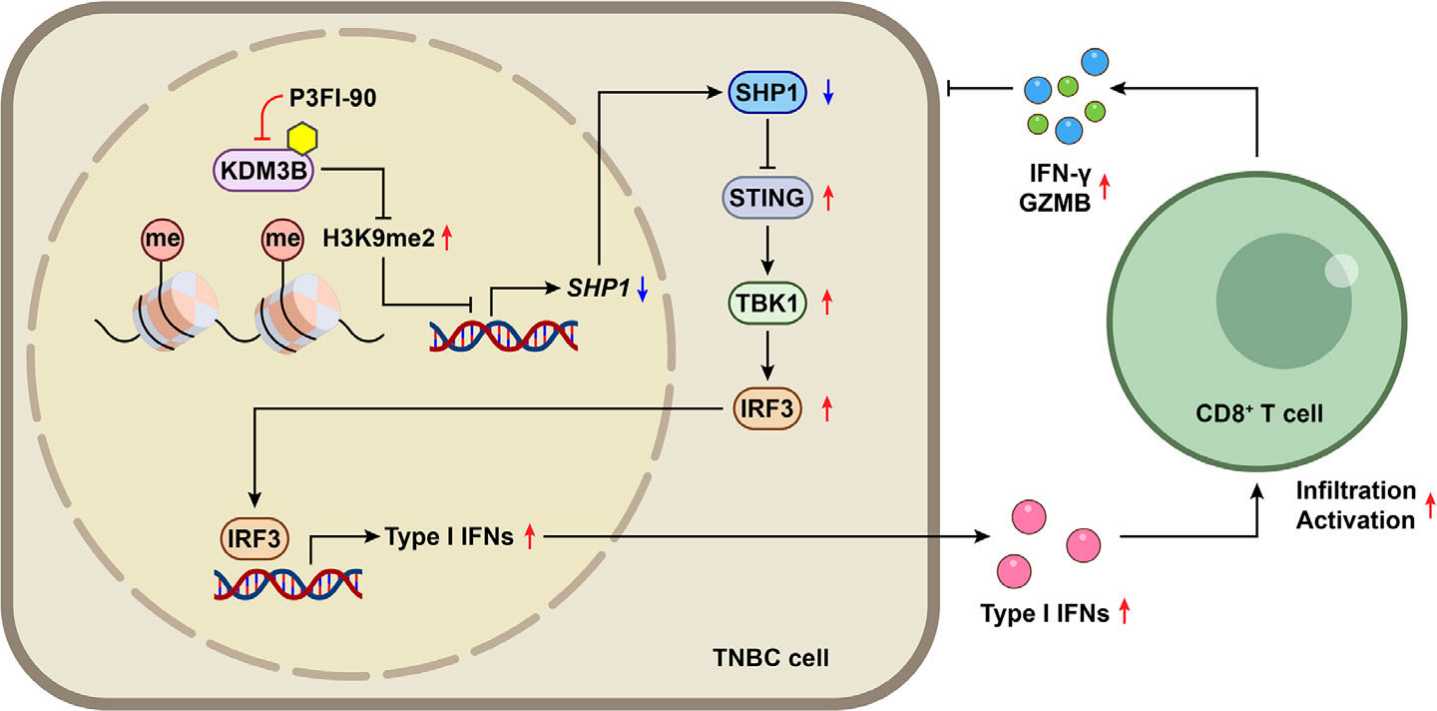
Schematic diagram illustrating the mechanism by which KDM3B regulates anti‐tumor immunity in TNBC. P3FI–90 treatment, targets KDM3B, reshapes the epigenetic landscape, and suppresses SHP1 expression, thereby activating STING–TBK1–IRF3–type I IFN signaling pathway. Consequently, CD8^+^ T cells are recruited to the tumor site and activated to produce IFN–γ and GZMB, leading to the killing of TNBC cells. IFN, interferon; GZMB, granzyme B.

In summary, the results of the present study reveal a tumor–intrinsic function of KDM3B in immune escape, particularly through the regulation of CD8^+^ T cell–mediated anti‐tumor immunity in TNBC. We demonstrate that genetic and pharmacologic inhibition of KDM3B triggers STING–mediated type I IFN responses by downregulating SHP1 expression, thereby promoting CD8^+^ T cell recruitment and activation. Therefore, targeting KDM3B represents a promising therapeutic strategy to enhance the efficacy of ICB therapy for TNBC (Figure 9).

## 4 Experimental Section

### 4.1 Cell Lines and Cell Culture

The human embryonic kidney cell line HEK293T (RRID: CVCL_0063) and murine TNBC cell lines 4T1 (RRID: CVCL_0125) and EMT6 (RRID: CVCL_1923) were purchased from American Type Culture Collection (ATCC). All cell lines were authenticated by short tandem repeat (STR) profiling and confirmed to be free of mycoplasma and bacterial contamination. The cell lines were maintained in DMEM (Macgene) supplemented with 10% fetal bovine serum (FBS; Gibco) and 1% penicillin–streptomycin (Macgene). The cells were cultured in a 5% CO_2_ incubator at 37°C.

### 4.2 Patient Samples

Tumors and adjacent normal tissues were collected from patients diagnosed with TNBC at Qilu Hospital. The tissues were obtained during surgery and preserved at −80°C until use. Follow–up data were available for all patients. This study was approved by the Ethics Committee on Scientific Research of Qilu Hospital of Shandong University, and informed consent was obtained from all patients. The approval number was KYLL‐2024(ZM)‐307.

### 4.3 Antibodies and Inhibitors

For flow cytometry, PE/Cyanine7 anti–mouse CD45 (Cat# 103114, BioLegend), PE anti–mouse CD3 (Cat# 100206, BioLegend), APC anti–mouse CD8a (Cat# 100712, BioLegend), FITC anti–mouse IFN–γ (Cat# 505806, BioLegend), and PerCP anti–human/mouse Granzyme B (Cat# 396416, BioLegend) were used. For the IHC assay, anti–CD8 alpha (Cat# ab217344, Abcam) was used. For western blotting, anti–H3K9me2 (Cat# ab1220, Abcam), and anti–TLR3 (Cat# ab307442, Abcam), anti–Histone H3 (Cat# 2650S, Cell Signaling Technology), anti–KDM3B (Cat# 5377T, Cell Signaling Technology), anti–p–TBK1 (Cat# 5483T, Cell Signaling Technology), anti–p–IRF3 (Cat# 4947S, Cell Signaling Technology), anti–IRF3 (Cat# 4302T, Cell Signaling Technology), anti–p–STING (Cat# 72971T, Cell Signaling Technology), anti–SHP1 (Cat# 3759S, Cell Signaling Technology), anti–MAVS (83000T, Cell Signaling Technology), anti–cGAS (Cat# 31659T, Cell Signaling Technology), anti–GAPDH (Cat# 60004–1–Ig, Proteintech), anti–STING (Cat# A3575, Abclonal), and anti–TBK1 (Cat# A3458, Abclonal) were used. For co–Immunoprecipitation (co–IP) assays, anti–STING (Cat# A3575, Abclonal) was used. For ChIP assays, anti–H3K9me2 (Cat# ab1220, Abcam) and anti–KDM3B (Cat# MA5–45972, Invitrogen) were used. For animal experiments, anti–mouse CD8α (Cat# BE0004–1, Bio X Cell), anti–mouse IFNAR–1 (Cat# 127323, BioLegend), and anti–PD–1 (Cat# BP0273, Bio X Cell) were used. The following inhibitors were purchased: P3FI–90 (Cat# HY–139348, MCE), SC–43 (Cat# HY–136657, MCE), RU.521 (Cat# HY–114180, MCE), and H–151 (Cat# HY–112693, MCE).

### 4.4 Stable Gene Knockdown and Transfection

For stable knockdown of *Kdm3b*, sh*Kdm3b* plasmids were obtained by cloning shRNA oligos targeting *Kdm3b* into the pLKO.1 empty vector. For stable knockdown of *Sting*, the pLV3–U6–Sting1–shRNA1–mCherry–Neo plasmid (Cat# P68186) was purchased from MiaoLingPlasmid. The plasmids, together with psPAX2 and pMD2.G, were co–transfected into HEK293T cells. The lentivirus–containing supernatant was collected by filtration through a 0.45 µm filter 48 h after transfection. Infected 4T1 and EMT6 cells were selected with puromycin at 5 µg/mL or G418 at 600 µg/mL. The sequences of shRNA oligos targeting *Kdm3b* and *Sting* are listed in Table S1. siRNAs were synthesized and purchased from Tsingke Biotech. The specific sequences against *Sting*, *Mavs*, *Tlr3*, and *Cgas* are listed in Table S1. Plasmids for *Shp1* and *Kdm*s overexpression were obtained from MiaoLingPlasmid. Plasmids for RNAi–resistant *Kdm3b*, or demethylase activity–deficient *Kdm*s were constructed using a KOD–Plus–Mutagenesis Kit (Cat# SMK–101, TOYOBO). All plasmids and siRNAs were transfected using Lipofectamine 2000 (Cat# 11668019, Invitrogen).

### 4.5 Animal Experiments

Six–week–old female BALB/c nude mice and BALB/c mice were purchased from Vital River Laboratory Animal Technology and were randomly assigned to groups. Mice were subcutaneously implanted with 5 × 10^5^ EMT6 vector control, stable knockdown, or WT cells in 100 µL of PBS. Tumor growth was measured every 2–3 days, and tumor volume was calculated using the formula: volume = 0.5 × length × width^2^. For KDM3B inhibitor (P3FI–90) treatment, P3FI–90 (20 mg/kg) was administered intraperitoneally (i.p.) daily for 7 days starting 5 days after tumor implantation. For CD8^+^ T cell and type I IFN depletion, 100 µg of CD8α or IFNAR1 neutralizing antibodies was injected i.p. on days 0, 4, 8, 12 after tumor implantation. For PD–1 blockade, 100 µg of anti–PD–1 antibody was injected i.p. on days 7, 10, 13 after tumor implantation. Mice were euthanized by carbon dioxide asphyxiation at the indicated timepoints, and tumor tissues were harvested for further analysis. All animal experiments were approved by the Institutional Animal Care and Use Committee of Qilu Hospital of Shandong University.

### 4.6 Flow Cytometry Analysis

Harvested tumor tissues were cut into pieces, digested, and filtered to obtain single–cell suspensions. Cells were stained with surface antibodies against CD45, CD3, and CD8 in the dark at 4°C for 30 min. After fixation and permeabilization, intracellular markers (IFN–γ and GZMB) were stained according to the manufacturer's instructions. Samples were analyzed on a flow cytometer (Accuri C6 plus, BD Biosciences Pharmingen), and data were analyzed using FlowJo software.

### 4.7 IHC Assay

Tumor sections were deparaffinized in xylene and rehydrated in graded ethanol. Following antigen retrieval and blocking of endogenous peroxidase activity, the sections were incubated with a primary antibody against mouse CD8α at 4°C overnight. The next day, the sections were incubated with a secondary antibody, stained with diaminobenzidine (DAB), and counterstained with hematoxylin. Immunostaining was visualized using an Olympus light microscope.

### 4.8 RNA Extraction and RT–qPCR

Total RNA was extracted from cancer cells using the RNA–easy Isolation Reagent (Cat# R701, Vazyme) according to the manufacturer's protocol. The extracted RNA was then reverse–transcribed into cDNA using an Evo M–MLV RT Kit (Cat# AG11707, AG). SYBR Green qPCR mix (Cat# 11201ES08, YEASEN) was used for PCR amplification. *GAPDH* (for human cells) or *Gapdh* (for murine cells) was used as an endogenous control. The gene–specific primers are listed in Table S2.

### 4.9 Western Blot Analysis

Cells were lysed in RIPA buffer (Cat# P0038, Beyotime) containing protease and phosphatase inhibitors. The protein concentration of each sample was determined using a BCA Protein Assay Kit (Cat# 71285–3, Millipore). Protein samples were then mixed with SDS loading buffer and boiled. Equal amounts of proteins were loaded onto SDS–PAGE gels and transferred to PVDF membranes (Millipore). The membranes were blocked with 5% non–fat milk and incubated with the indicated primary antibodies at 4°C overnight. The next day, after being incubation with secondary antibody for 2 h, the immunoblotting bands were visualized using enhanced chemiluminescence (ECL) reagent.

### 4.10 Co–Immunoprecipitation (co–IP) Assay

EMT6 cells were lysed in Western and IP lysis buffer (Cat# P0013, Beyotime), and the lysates were divided into input and IP groups. Anti–STING antibody or control IgG was added to the IP groups, and the protein–antibody mixtures were rotated at 4°C overnight. The next day, Protein A/G Magnetic Beads (Cat# HY–K0202, MCE) were added to the mixtures and incubated with rotation for an additional 2 h. After washing, the magnetic beads were collected. Following boiling with SDS loading buffer, the supernatants were subjected to Western blot analysis.

### 4.11 RNA–seq and Functional Annotation

Total RNA from DMSO– or P3FI–90–treated 4T1 cells was extracted using TRIzol reagent (Cat # 15596026CN, Invitrogen). Transcriptome libraries were prepared using the Illumina mRNA–Seq Sample Preparation Kit (San Diego, CA). The libraries were qualified and quantified using an Agilent 2100 bioanalyzer (Thermo Fisher Scientific) and then sequenced on the Illumina Novaseq 6000 platform (Tsingke Biotech). DEGs between the DMSO and P3FI–90 groups were identified using the “limma” R package and subjected to the Database for Annotation, Visualization and Integrated Discovery (DAVID) website (https://david.ncifcrf.gov/) for GO and KEGG analyses. GSEA was performed using GSEA 4.3.3 software.

### 4.12 ATAC–seq

A total of 50, 000 control or KDM3B–inhibited cells (sh*Kdm3b* or P3FI–90, 10 µm, 24 h) were centrifuged, washed once with cold PBS, and resuspended in cold lysis buffer. After removal of the supernatant, the cell nuclei were resuspended in the transposition reaction system containing Tn5 Transposase, and the DNA was purified after incubation at 37°C for 30 min. The PCR reaction mixture was prepared with the purified DNA, and PCR amplification was performed. The final DNA libraries were sequenced on an Illumina NovaSeq X Plus platform (Tsingke Biotech) after the DNA purification. Reads were trimmed to remove adaptor sequences using the Cutadapt software to obtain high–quality reads in FASTQ format. Bowtie2 software was used to align the high–quality reads to the mouse mm10 genome. DeepTools v2.0.7 was used to map the read density distribution in the ± 3 kb regions around the transcription start site (TSS) of each gene. A sliding window method was used to statistically analyze read abundance across the whole genome. ATAC–seq–enriched picks were identified using MACS2 v2.1.1. Aligned reads were visualized using the Integrative Genomics Viewer (IGV) [44].

### 4.13 ChIP–qPCR

ChIP assays were performed using an Enzymatic ChIP Assay Kit (Cat# P2083S, Beyotime) according to the manufacturer's protocol. Enrichment of H3K9me2 at the *Shp1* promoter was detected by RT–qPCR. The sequences of the *Shp1* promoter are listed in Table S2.

### 4.14 Methylthiazolyldiphenyl–tetrazolium Bromide (MTT) Assay

A total of 500 TNBC cells were seeded in 96–well plates. The plates were then incubated with MTT for 4 h. After replacing the supernatants with DMSO, the absorbance values at 490 nm were measured.

### 4.15 Colony Formation Assay

A total of 500 TNBC cells were seeded in 6–well plates and cultured for 7 days. Colonies were fixed with methanol and stained with crystal violet. The number of colonies was counted using ImageJ software.

### 4.16 Statistical Analysis

All experiments were independently repeated at least three times. All statistical analyses were performed using GraphPad Prism 8 and R version 4.4.2. Data are presented as mean ± SD. The Kaplan–Meier method was used to assess overall survival. Unpaired two–tailed Student's *t*–tests were used to compare differences between two groups. Two–way analysis of variance (ANOVA) was used for multiple comparisons in MTT assays and tumor growth experiments. A *p*‐value of < 0.05 was considered statistically significant (^*^ *p* < 0.05, ^**^ *p* < 0.01, ^***^ *p* < 0.001, ^****^ *p* < 0.0001, and ns means not significant).

## Author Contributions

**Xiaolong Wang** and **Wenhao Li** contributed equally to this work. Q. Y., X. W., and W. L. conceived and designed the study. X. W., W. L., and Y. W. performed the experiments. B. C., W. Z., L. W., and D. L. collected clinical samples. X. W., W. L., and N. Z. analyzed the data. X. W. and W. L. wrote the paper. Q. Y. and X. W. revised the paper. All authors read and approved the final manuscript.

## Ethics Statement

All human studies were approved by the Ethics Committee on Scientific Research of Qilu Hospital of Shandong University (Approval No. KYLL‐2024(ZM)‐307), and informed consent was obtained from all patients. All animal experiments were approved by the Institutional Animal Care and Use Committee of Qilu Hospital of Shandong University (Approval No. DWLL‐202400136).

## Consent

All authors have reviewed and approved the final version of the manuscript for publication.

## Conflicts of Interest

The authors declare no conflicts of interest.

## Supporting information




**Supporting file**: advs75846‐sup‐0001‐SuppMat.docx

## Data Availability

The data that support the findings of this study are available from the corresponding author upon reasonable request.
