# Supplementary material for: Targeting KDM3B Elicits Anti‐tumor Immunity by Alleviating SHP1–mediated STING Suppression in Triple–Negative Breast Cancer
Source: Adv Sci (Weinh). 2026 May 27:e75846. Online ahead of print. doi: 10.1002/advs.75846 (PMC13335874; doi:10.1002/advs.75846)
Supplement: Supplementary file 1 — Supporting file: advs75846‐sup‐0001‐SuppMat.docx [file ADVS-9999-e75846-s001.docx]

**Supplementary materials for**

**Targeting KDM3B elicits antitumor immunity by alleviating SHP1–mediated STING suppression in triple–negative breast cancer**

Xiaolong Wang^1#^, Wenhao Li^1#^, Yifei Wang^1^, Ning Zhang^1^, Bing Chen^2^, Wenjing Zhao^2^, Lijuan Wang^2^, Dan Luo^2^, Qifeng Yang^1,2,3*^

^*^**Address correspondence to:** Qifeng Yang (qifengy_sdu@163.com).

**
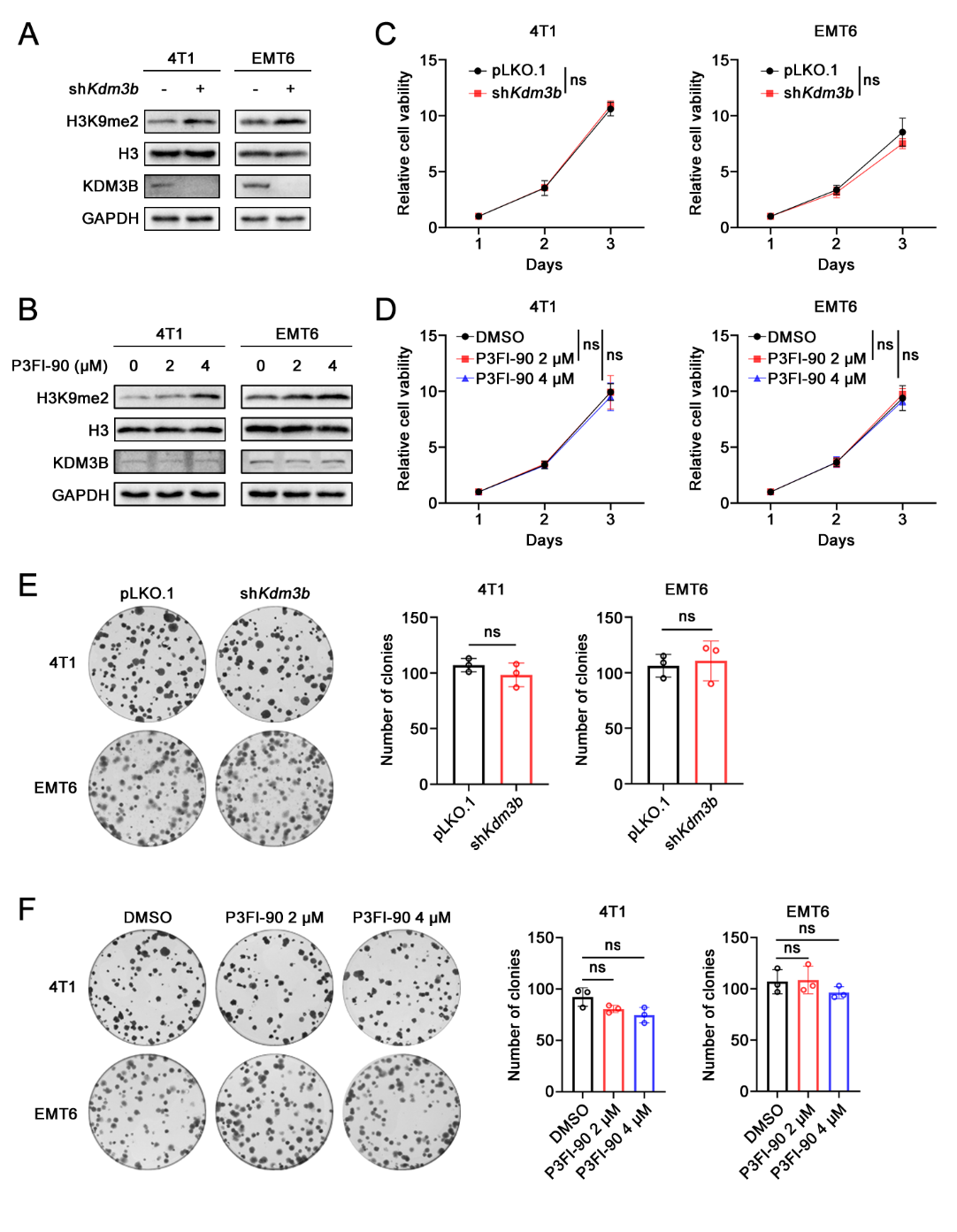
**

**Figure S1. Inhibition of** **KDM3B does not affect TNBC cell viability.**

A, B) Effects of KDM3B inhibition (sh*Kdm3b* or P3FI–90, 24 h) on H3K9me2 modification and KDM3B expression detected by Western blotting in TNBC cells.

C–F) Effects of KDM3B inhibition (sh*Kdm3b* or P3FI–90, 24 h) on cell viability detected by MTT and colony formation assays in TNBC cells (C, D: MTT; E, F: colony formation).

Data are presented as means ± SD. Two–way ANOVA in (C, D). Unpaired two–tailed Student’s t–test in (E, F). ns, not significant.


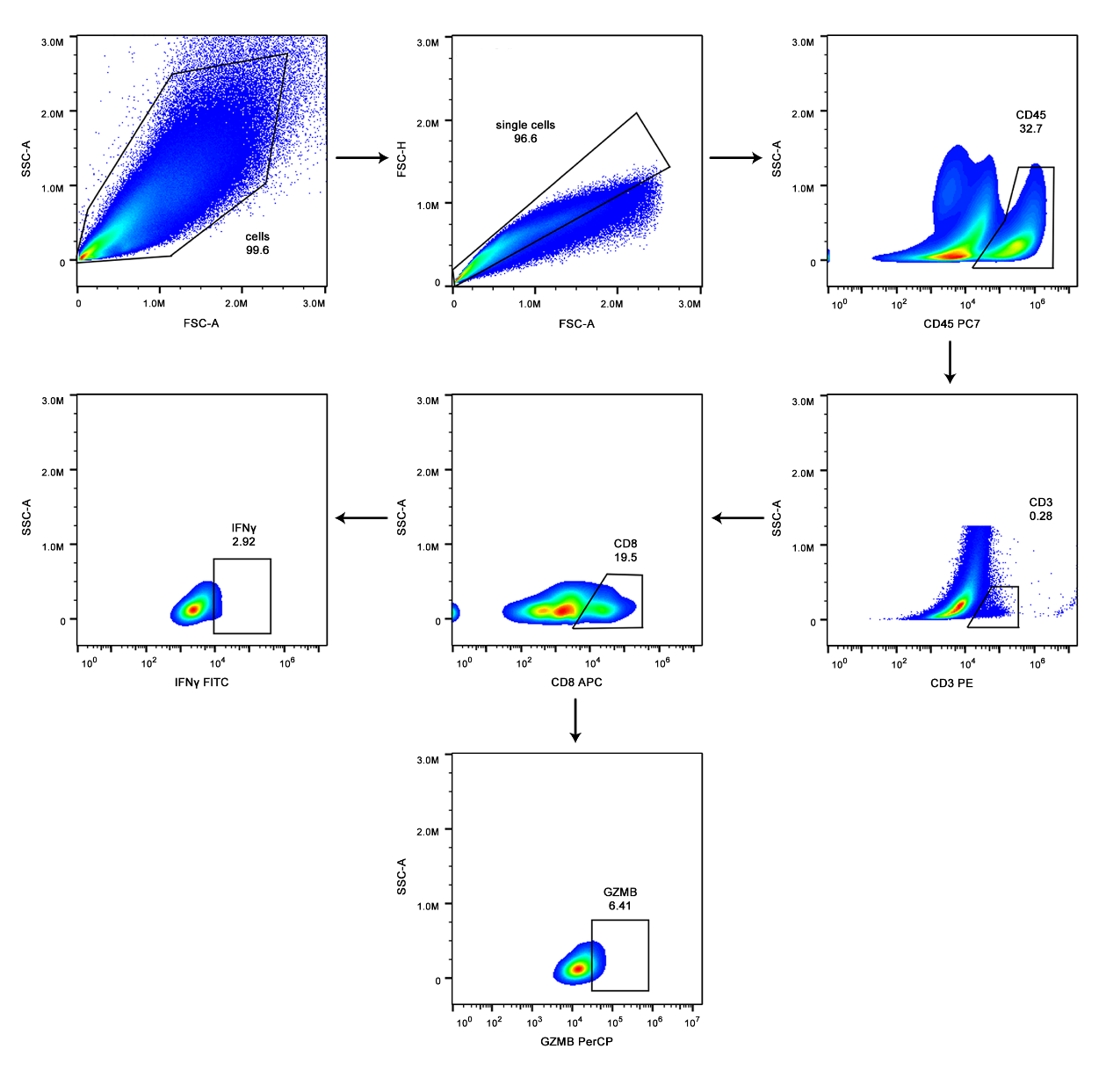


**Figure S2. Representative gating strategy for identifying IFN–γ^+^CD8^+^ and GZMB^+^CD8^+^ T cells.**


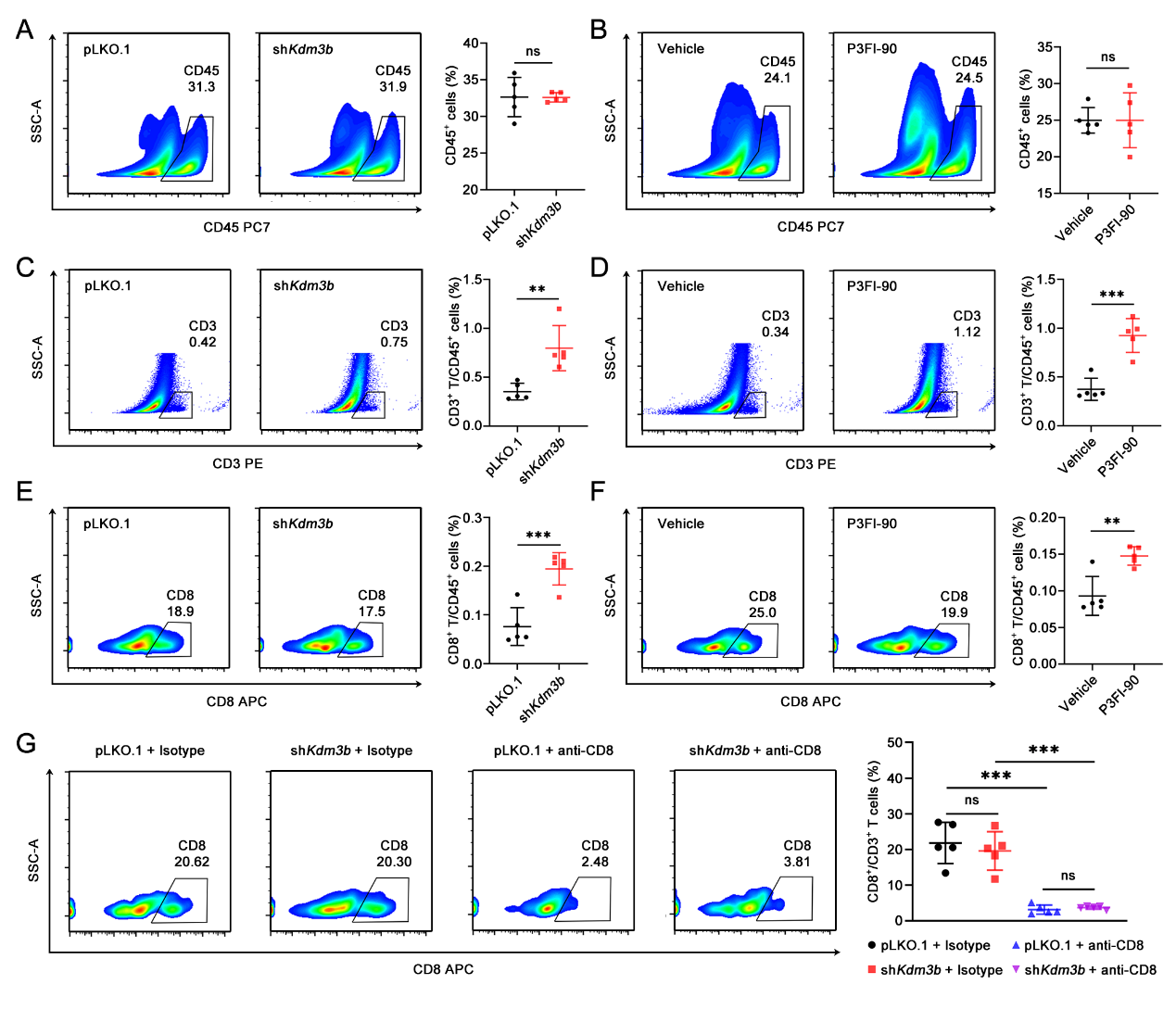


**Figure S3. Quantification of immune cell populations following KDM3B inhibition or anti–CD8 treatment.**

A, B) Quantification of CD45^+^ cells in EMT6 xenografts treated with KDM3B inhibition (sh*Kdm3b* or P3FI–90).

C, D) Quantification of CD3^+^ T cells in EMT6 xenografts treated with KDM3B inhibition (sh*Kdm3b* or P3FI–90).

E, F) Quantification of CD8**^+^** T cells in EMT6 xenografts treated with KDM3B inhibition (sh*Kdm3b* or P3FI–90).

G) Quantification of CD8**^+^** T cells in pLKO.1 or sh*Kdm3b* EMT6 xenografts treated with control IgG or anti–CD8α antibody.

Data are presented as means ± SD. Unpaired two–tailed Student’s t–test in (A–G). ns, not significant; ***P* < 0.01, ****P* < 0.001.


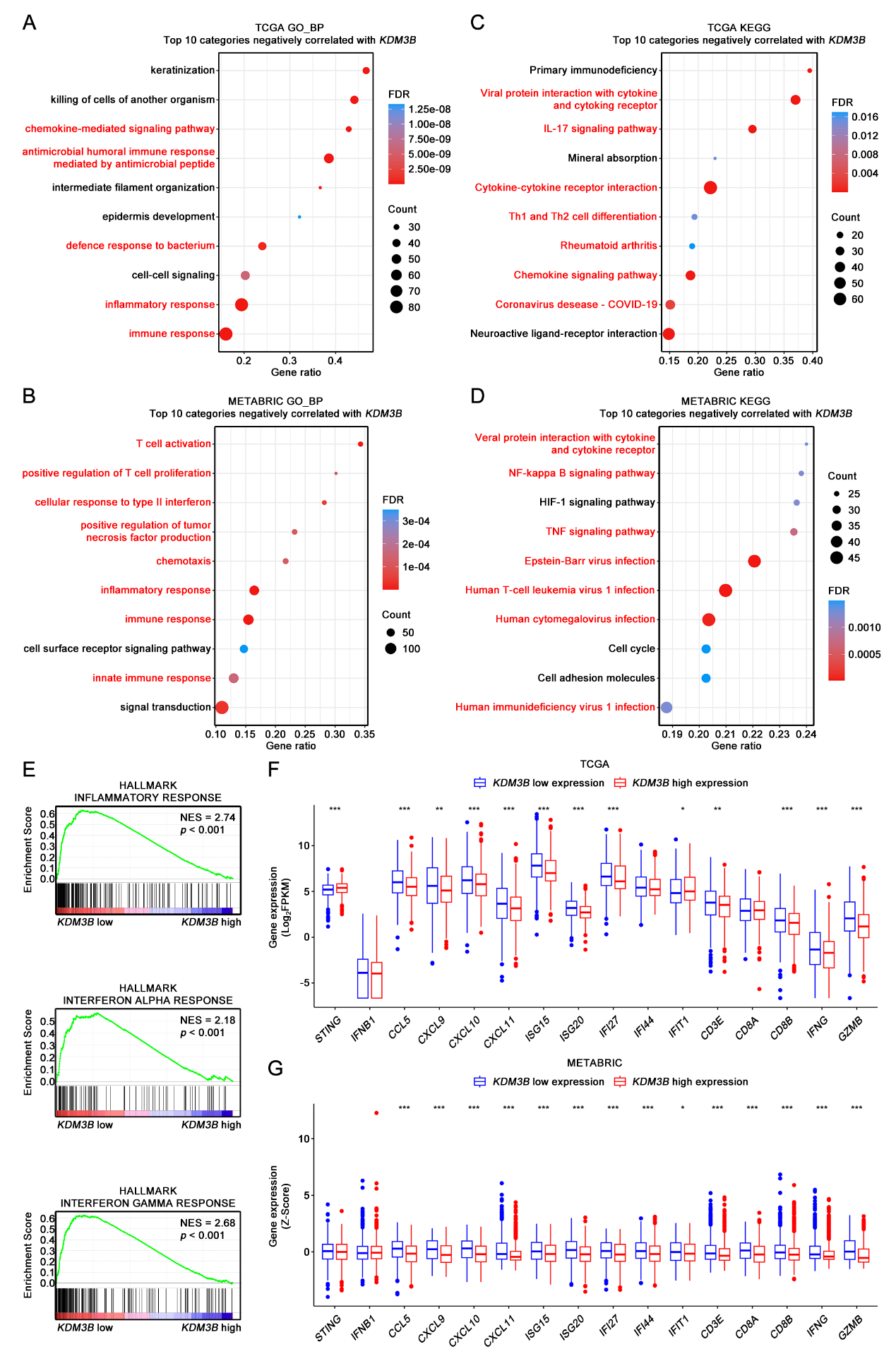


**Figure S4. KDM3B is negatively correlated with type Ⅰ IFN responses in human breast cancers.**

A, B) Gene ontology (GO) analyses identifying the top 10 pathways negatively correlated with *KDM3B* expression in the TCGA (A) and METABRIC (B) databases.

C, D) Kyoto encyclopedia of genes and genomes (KEGG) analyses identifying the top 10 pathways negatively correlated with *KDM3B* expression in the TCGA (C) and METABRIC (D) databases.

E) Gene set enrichment analysis (GSEA) identifying three hallmark signatures negatively correlated with *KDM3B* expression in the METABRIC database. NES, normalized enrichment score.

F, G) Expression levels of the indicated genes in *KDM3B* low– versus high–expression groups in the TCGA (F) and METABRIC (G) databases.

Unpaired two–tailed Student’s t–test in (F, G). **P* < 0.05, ***P* < 0.01, ****P* < 0.001.


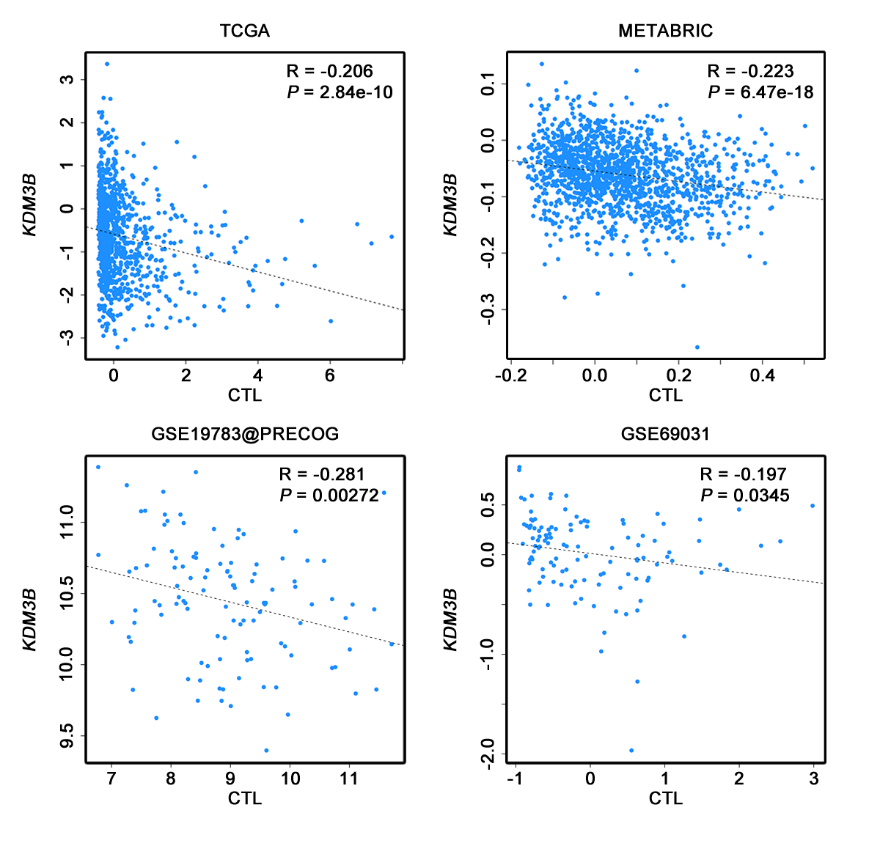


**Figure S5. Correlation between KDM3B expression and cytotoxic T lymphocyte (CTL) infiltration.** Analysis was conducted following the TIDE methodology.^1^ *Note: The original TIDE web server (*[*http://tide.dfci.harvard.edu/*](http://tide.dfci.harvard.edu/)*) has been retired and replaced by the Cancer Immunology Data Engine (CIDE; https://cide.ccr.cancer.gov).*


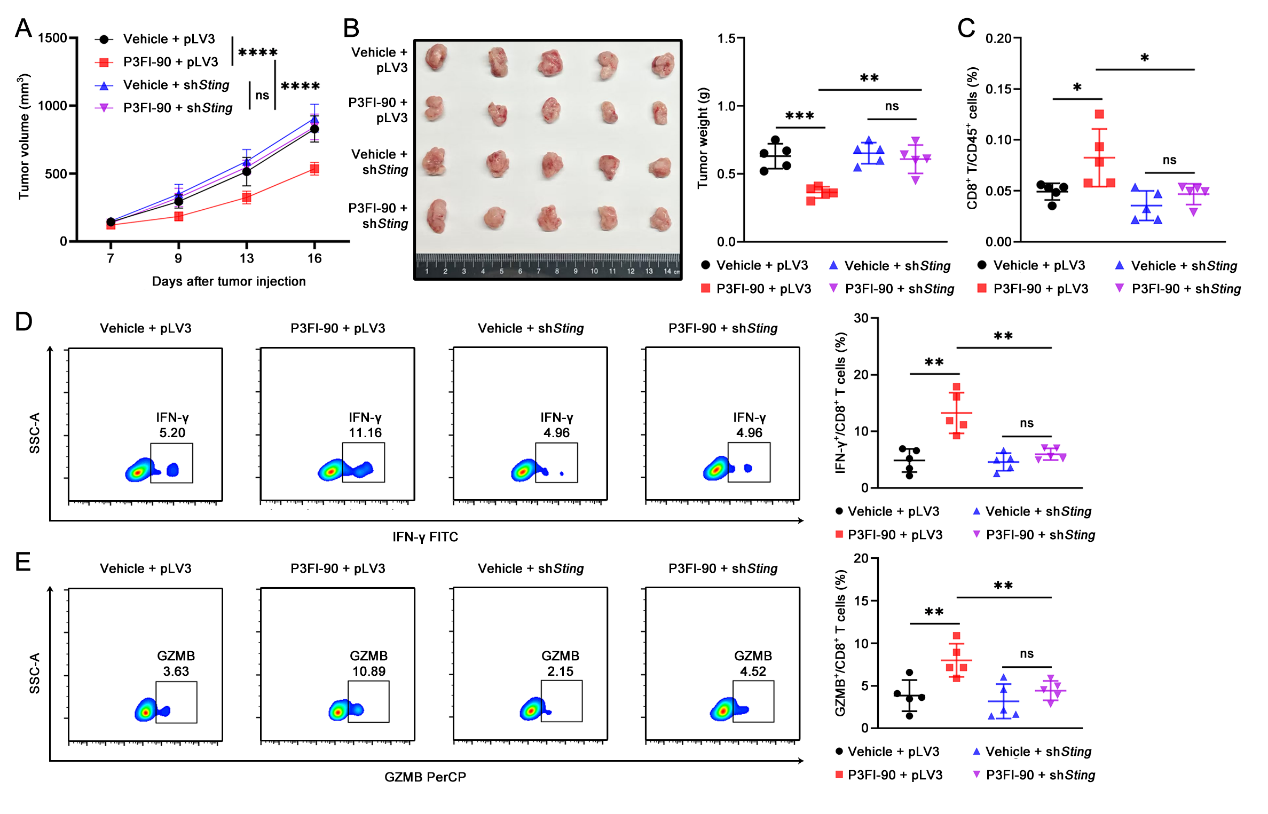


**Figure S6. P3FI–90–induced antitumor immunity is dependent on tumor–intrinsic STING.**

A, B) Tumor growth curves, tumor image, and tumor weight curves of EMT6 xenografts treated with P3FI–90 or sh*Sting* (A, growth curves; B, tumor images and tumor weight).

C) Quantification of CD8**^+^** T cells in pLV3 or sh*Sting* EMT6 xenografts treated with P3FI–90.

D) Quantification of IFN–γ^+^CD8^+^ T cells in pLV3 or sh*Sting* EMT6 xenografts treated with P3FI–90.

E) Quantification of GZMB^+^CD8^+^ T cells in pLV3 or sh*Sting* EMT6 xenografts treated with P3FI–90.

n = 5 mice per group. Data are presented as means ± SD. Two–way ANOVA in (A). Unpaired two–tailed Student’s t–test in (C–E). ns, not significant; **P* < 0.05, ***P* < 0.01, ****P* < 0.001, *****P* < 0.0001.


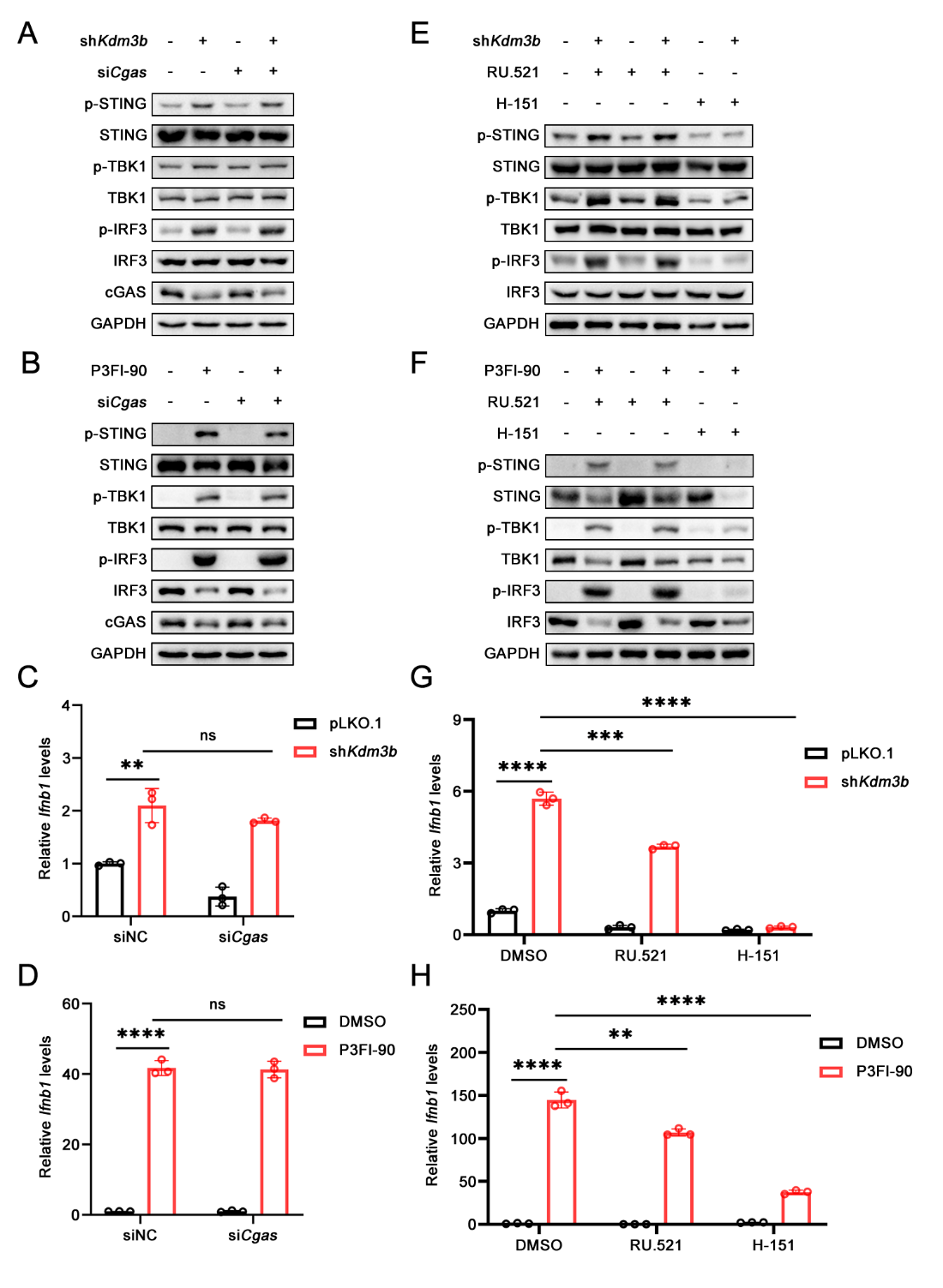


**Figure S7. Inhibition of KDM3B triggers STING–type Ⅰ IFN signaling in a cGAS–independent manner.**

A, B) Effects of KDM3B inhibition (sh*Kdm3b* or P3FI–90, 10 μM, 24 h) or *Cgas* knockdown on STING, TBK1, and IRF3 activation detected by Western blotting in EMT6 cells.

C, D) Effects of KDM3B inhibition (sh*Kdm3b* or P3FI–90, 10 μM, 24 h) or *Cgas* knockdown on *Ifnb1* mRNA expression levels detected by RT–qPCR in EMT6 cells.

E, F) Effects of KDM3B inhibition (sh*Kdm3b* or P3FI–90, 10 μM, 24 h), RU.521 (10 μM, 24 h), or H–151 (10 μM, 24 h) treatment on STING, TBK1, and IRF3 activation detected by Western blotting in EMT6 cells.

G, H) Effects of KDM3B inhibition (sh*Kdm3b* or P3FI–90, 10 μM, 24 h), RU.521(10 μM, 24 h), or H–151(10 μM, 24 h) treatment on *Ifnb1* mRNA expression levels detected by RT–qPCR in EMT6 cells.

Data are presented as means ± SD. Unpaired two–tailed Student’s t–test in (C, D, G, H). ns, not significant; ***P* < 0.01, ****P* < 0.001, *****P* < 0.0001.


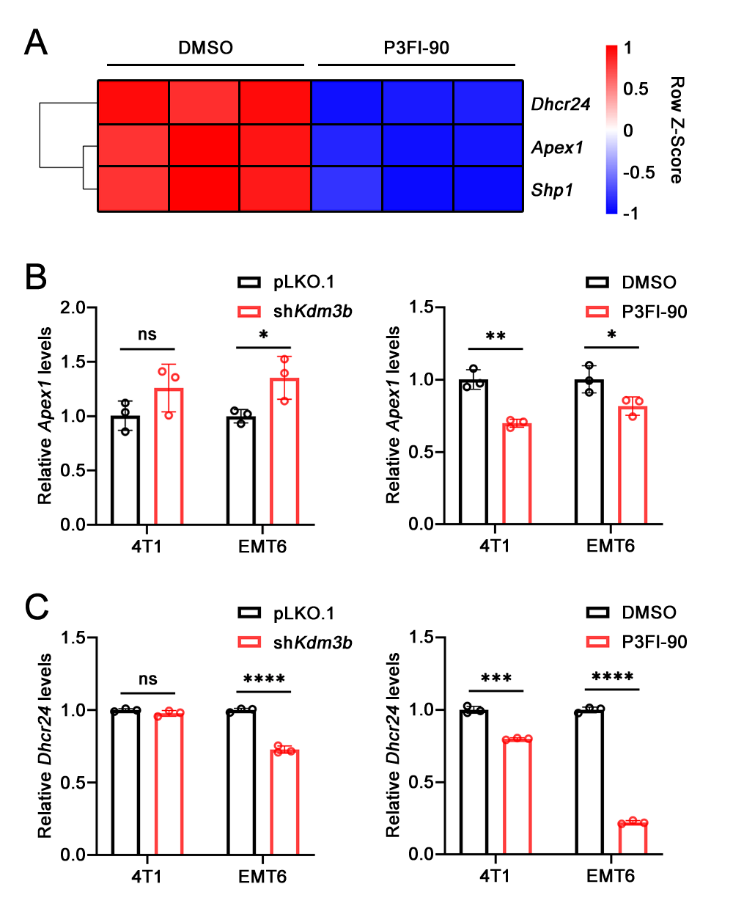


**Figure S8. Inhibition of KDM3B targets SHP1.**

A) Heatmap showing differential expression of *Dhcr24*, *Apex1*, and *Shp1* between DMSO– and P3FI–90–treated 4T1 cells.

B, C) Expression of *Apex1* (B) and *Dhcr24* (C) measured by RT–qPCR following KDM3B inhibition (sh*Kdm3b* or P3FI–90, 10 μM, 24 h) in TNBC cells.

Data are presented as means ± SD. Unpaired two–tailed Student’s t–test in (B, C). ns, not significant; **P* < 0.05, ***P* < 0.01, ****P* < 0.001, *****P* < 0.0001.


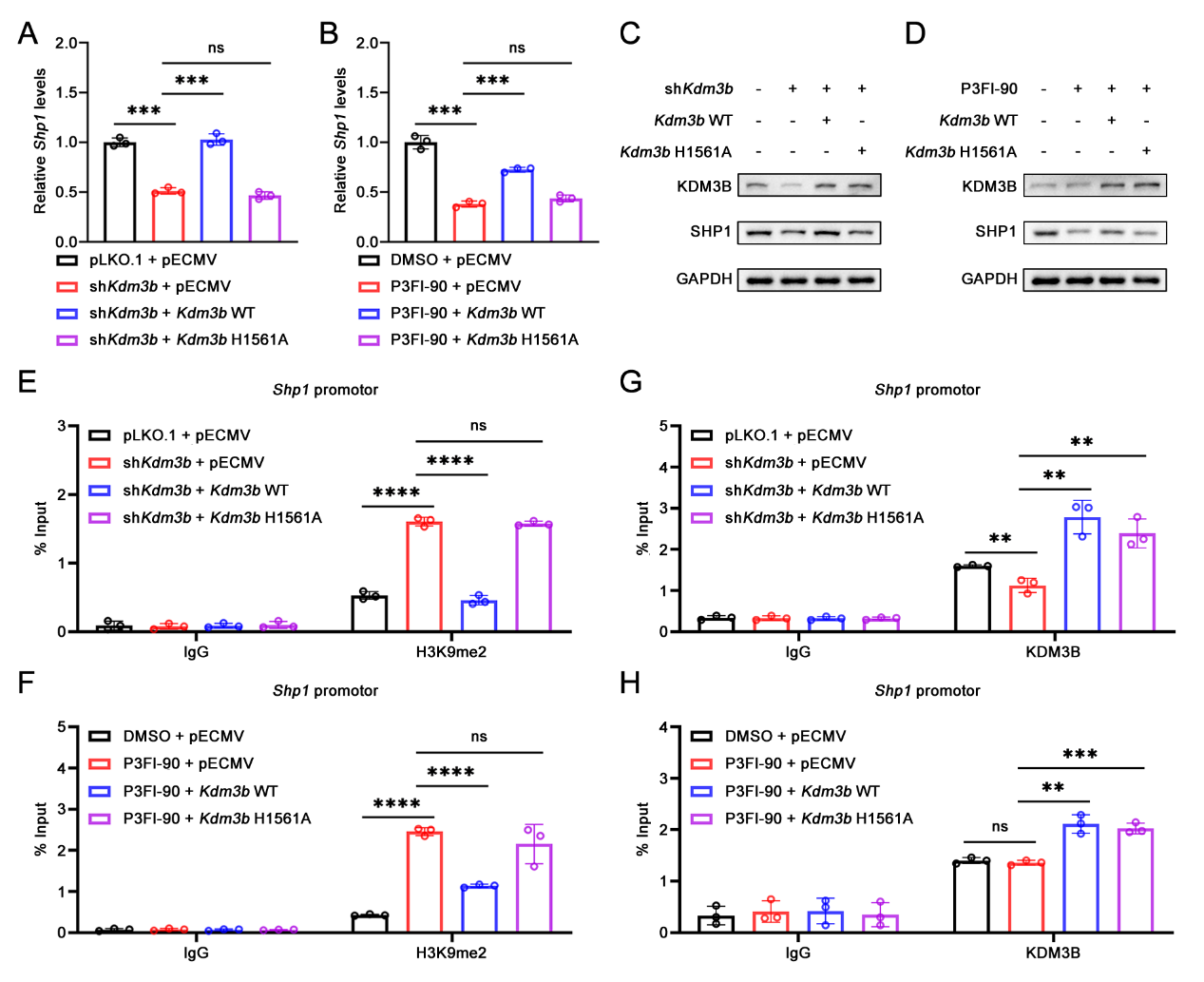


**Figure S9. KDM3B regulates SHP1 expression through its demethylase activity.**

A, B) Effects of KDM3B inhibition (sh*Kdm3b* or P3FI–90, 10 μM, 24 h) or *Kdm3b* overexpression (wild–type [WT] or H1561A mutant) on *Shp1* mRNA expression measured by RT–qPCR with in EMT6 cells.

C, D) Effects of KDM3B inhibition (sh*Kdm3b* or P3FI–90, 10 μM, 24 h) or *Kdm3b* overexpression (WT or H1561A) on SHP1 protein expression detected by Western blotting in EMT6 cells.

E, F) ChIP–qPCR assays assessing H3K9me2 enrichment at the *Shp1* promotor following KDM3B inhibition (sh*Kdm3b* or P3FI–90, 10 μM, 24 h) or *Kdm3b* overexpression (WT or H1561A) in EMT6 cells.

G, H) ChIP–qPCR assays assessing KDM3B binding at the *Shp1* promotor following KDM3B inhibition (sh*Kdm3b* or P3FI–90, 10 μM, 24 h) or *Kdm3b* overexpression (WT or H1561A) on in EMT6 cells.

Data are presented as means ± SD. Unpaired two–tailed Student’s t–test in (A, B, E–H). ns, not significant; ***P* < 0.01, ****P* < 0.001, *****P* < 0.0001.


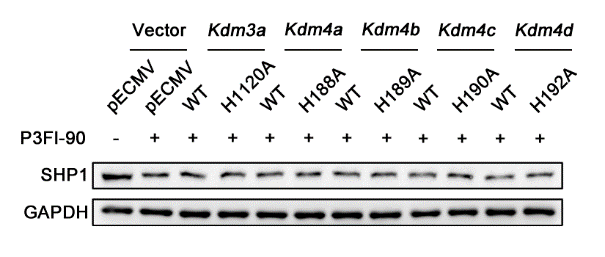


**Figure S10. P3FI–90 reduces SHP1 protein expression independently of KDM3A or KDM4A–D.**

Effects of P3FI–90 treatment (10 μM, 24 h), or overexpression of wild–type or catalytically mutants of *Kdm3a*, *Kdm4a*, *Kdm4b*, *Kdm4c*, *Kdm4d* on SHP1 protein expression detected by Western blotting.


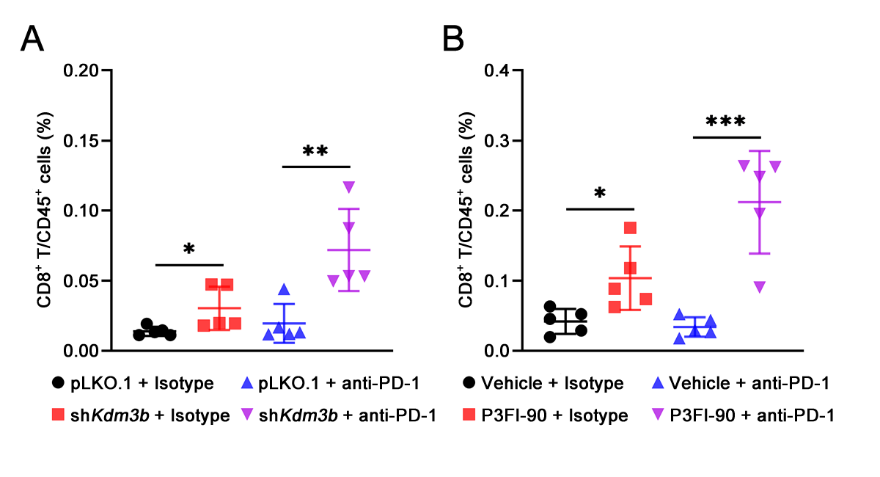


**Figure S11. Quantification of CD8^+^ T cells following KDM3B inhibition combined with anti–PD–1 therapy.**

A, B) Quantification of CD8^+^ T cells in EMT6 xenografts treated with KDM3B inhibition (sh*Kdm3b* or P3FI–90) combined with control IgG or anti–PD–1 antibody. Data are represented as means ± SD. Unpaired two–tailed Student’s t–test in (A, B). **P* < 0.05, ***P* < 0.01, ****P* < 0.001.


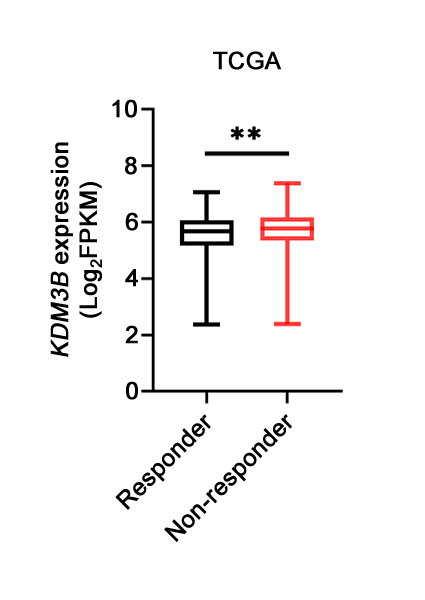


**Figure S12. KDM3B expression in immunotherapy responders versus non–responders in the TCGA database.** Analysis was conducted using the TIDE method.^1^ *Note: The original TIDE web server (*[*http://tide.dfci.harvard.edu/*](http://tide.dfci.harvard.edu/)*) has been retired and replaced by the Cancer Immunology Data Engine (CIDE; https://cide.ccr.cancer.gov).*

**References**

1. Jiang P*, et al.* Signatures of T cell dysfunction and exclusion predict cancer immunotherapy response. *Nature medicine* **24**, 1550–1558 (2018).

**Table S1. Sequences for the nucleic acids used in this study.**

| **Targets** | **Targeted sequences (5’–3’)** |
| --- | --- |
| **sh*Kdm3b*** | CAGAAGGTTCTGCGTCAAATA |
| **sh*Sting*** | ATGATTCTACTATCGTCTTAT |
| **si*Sting*** | TGATTTGGTGGATCCTTTG |
| **si*Mavs*** | GGCTGATCAAGTGACTCGA |
| **si*Tlr3*** | GCAGAAGATTCAAGGTACA |
| **si*Cgas*** | GCTGTAACACTTCTTATCA |

**Supplementary Table 2. Primers for RT–qPCR.**

| **Primer sets** | **Primers** | **Sequence (5’–3’)** |
| --- | --- | --- |
| **M–*Ifnb1*** | Forward | AGCTCCAAGAAAGGACGAACA |
|  | Reverse | GCCCTGTAGGTGAGGTTGAT |
| **M–*Ifit1*** | Forward | TACAGGCTGGAGTGTGCTGAGA |
|  | Reverse | CTCCACTTTCAGAGCCTTCGCA |
| **M–*Cxcl10*** | Forward | CTCATCCTGCTGGGTCTGAG |
|  | Reverse | CCTATGGCCCTCATTCTCAC |
| **M–*Ifi44*** | Forward | ATGCACTCTTCTGAGCTGGTGG |
|  | Reverse | TCAGATCCAGGCTATCCACGTG |
| **M–*Isg15*** | Forward | CATCCTGGTGAGGAACGAAAGG |
|  | Reverse | CTCAGCCAGAACTGGTCTTCGT |
| **M–*Ccl5*** | Forward | CCTGCTGCTTTGCCTACCTCTC |
|  | Reverse | ACACACTTGGCGGTTCCTTCGA |
| **M–*Shp1*** | Forward | TTGGCAGGAGAACACTCGTGTC |
|  | Reverse | TGCTCCCTACTGTTGGTCACAG |
| **M–*Apex1*** | Forward | ATGCTCCTGGAATGTGGATGGG |
|  | Reverse | GTTTGTTCTCCGAGCACTTGGTC |
| **M–*Dhcr24*** | Forward | CTGGAGAACCACTTCGTGGAAG |
|  | Reverse | CTCCACATGCTTGAAGAACCAGG |
| **M–*Gapdh*** | Forward | CATCACTGCCACCCAGAAGACTG |
|  | Reverse | ATGCCAGTGAGCTTCCCGTTCAG |
| **H–*KDM3B*** | Forward | CCCCAGGACCTAGCGATCTT |
|  | Reverse | AGCAGAAGCACGATAACTTCC |
| **H–*GAPDH*** | Forward | GTCTCCTCTGACTTCAACAGCG |
|  | Reverse | ACCACCCTGTTGCTGTAGCCAA |
| **M–*Shp1* promoter** | Forward | CCTCTGGTCTTGGCTTGGTAG |
|  | Reverse | TTATGGCCAGGGTCCCAACA |
